# Multiscale analysis of autotroph-heterotroph interactions in a high-temperature microbial community

**DOI:** 10.1371/journal.pcbi.1006431

**Published:** 2018-09-27

**Authors:** Kristopher A. Hunt, Ryan M. Jennings, William P. Inskeep, Ross P. Carlson

**Affiliations:** 1 Thermal Biology Institute, Montana State University, Bozeman, Montana, United States of America; 2 Center for Biofilm Engineering, Montana State University, Bozeman, Montana, United States of America; 3 Department of Chemical and Biological Engineering, Montana State University, Bozeman, Montana, United States of America; 4 Department of Land Resources and Environmental Sciences, Montana State University, Bozeman, Montana, United States of America; Ecole Polytechnique Fédérale de Lausanne, SWITZERLAND

## Abstract

Interactions among microbial community members can lead to emergent properties, such as enhanced productivity, stability, and robustness. Iron-oxide mats in acidic (pH 2–4), high-temperature (> 65 °C) springs of Yellowstone National Park contain relatively simple microbial communities and are well-characterized geochemically. Consequently, these communities are excellent model systems for studying the metabolic activity of individual populations and key microbial interactions. The primary goals of the current study were to integrate data collected *in situ* with *in silico* calculations across process-scales encompassing enzymatic activity, cellular metabolism, community interactions, and ecosystem biogeochemistry, as well as to predict and quantify the functional limits of autotroph-heterotroph interactions. Metagenomic and transcriptomic data were used to reconstruct carbon and energy metabolisms of an important autotroph (*Metallosphaera yellowstonensis*) and heterotroph (*Geoarchaeum* sp. OSPB) from the studied Fe(III)-oxide mat communities. Standard and hybrid elementary flux mode and flux balance analyses of metabolic models predicted cellular- and community-level metabolic acclimations to simulated environmental stresses, respectively. *In situ* geochemical analyses, including oxygen depth-profiles, Fe(III)-oxide deposition rates, stable carbon isotopes and mat biomass concentrations, were combined with cellular models to explore autotroph-heterotroph interactions important to community structure-function. Integration of metabolic modeling with *in situ* measurements, including the relative population abundance of autotrophs to heterotrophs, demonstrated that Fe(III)-oxide mat communities operate at their maximum total community growth rate (i.e. sum of autotroph and heterotroph growth rates), as opposed to net community growth rate (i.e. total community growth rate subtracting autotroph consumed by heterotroph), as predicted from the maximum power principle. Integration of multiscale data with ecological theory provides a basis for predicting autotroph-heterotroph interactions and community-level cellular organization.

## Author summary

Microbial communities often display emergent properties, such as enhanced productivity, stability, and robustness, compared to their component populations in isolation. However, determining the governing principles of these emergent properties can be elusive due to the complexities of interpreting and integrating genomic and geochemical data sets collected at largely different observational scales. Here, we use multiscale, metagenome-enabled modeling of an Fe(II)-oxidizing community to extract information regarding biomass productivity limitations, relative population abundance, total biomass concentration, and electron acceptor uptake rates. The systematic approach used herein is broadly applicable to any microbial community with modest activity and metagenomic data as well as provides a mechanism to characterize interaction motifs in communities that include uncultivated organisms.

## Introduction

Microorganisms are the largest component of the biosphere and drive biogeochemical cycles through metabolic activity [1]. Microorganisms commonly exist in biofilms or mats that contain numerous microenvironments due to the interplay between convection-, diffusion-, and chemical concentration gradients induced by microbial activity [2–4]. In addition, most natural microbial communities have diverse microbial populations and an array of nutrient and energy sources, which often precludes detailed analyses of microbial interactions linked to metabolic activity. Natural microbial communities containing well-characterized microbial populations and tractable nutrient inputs are excellent systems to elucidate the principles that organize microbial metabolism and interaction.

The phylogenetic diversity of microorganisms within Fe(III)-oxide microbial mats of acid-sulfate-chloride springs in Yellowstone National Park (YNP) is limited due to high temperature (65–75°C) and low pH (~ 3) [5–7]. These biomineralizing communities are formed and inhabited by a limited number of distinct phylotypes, including crenarchaea from the order Sulfolobales (e.g. *Metallosphaera yellowstonensis* str. MK1) and candidate phylum Geoarchaeota (e.g. *Geoarchaeum* str. OSPB) (supplemental material) [6–12]. The aqueous and solid-phase geochemistry of two such environments in Beowulf and One Hundred Springs Plain (OSP) hot springs have been studied in detail [5,12–15], and provide bounding conditions and physicochemical context for modeling microbial community interactions. The primary electron donors that drive chemolithoautotrophy in Fe(III)-oxide microbial mats include Fe(II) (25–40 μM) and possibly reduced forms of sulfur (dissolved sulfide < 10 μM) and As(III) (25–30 μM) [9]. The oxidation of Fe(II) coupled with the reduction of oxygen provides energy necessary for the fixation of carbon dioxide by *M*. *yellowstonensis*, a major autotroph in these mats [6,8,14,16,17]. The consumption of oxygen is diffusion-limited in Fe(III)-oxide microbial mats, and results in steep gradients in dissolved oxygen from 60 μM to below 1 μM over 0.5 to 1 mm [14]. The steep oxygen concentration gradients and corresponding relative abundance of community members as a function of mat depth indicate microbial competition for this limiting electron acceptor [12]. Genomic and mRNA data indicate that predominant autotrophs (e.g. *M*. *yellowstonensis*) and heterotrophs (e.g. *Geoarchaeum* str. OSPB) in Fe(III)-oxide mats utilize oxygen as an electron acceptor [7,16,18].

Carbon dioxide fixation by autotrophs in the community contributes 42 to 99% of the total microbial biomass carbon in Fe(III)-oxide mats from Beowulf and OSP hot springs; the remaining carbon originates from exogenous sources that are produced independent of system electron donor and acceptor requirements [17]. Carbon dioxide fixation has been demonstrated in *M*. *yellowstonensis*, which is one of the primary autotrophs in the oxic zones of the Fe(III)-oxide mats [17,19]. Metagenome analysis has established *Geoarchaeum* str. OSPB as a primary aerobic heterotroph, which comprises 30 to 50% of the total microbial community in the oxic zones of Fe(III)-oxide mats found at OSP [7,12]. Chemolithoautotrophic metabolism and the subsequent transfer of nutrients and energy to heterotrophs (e.g. *Geoarchaeum* str. OSPB) is hypothesized to drive major autotroph-heterotroph interactions along with competition for the primary terminal electron acceptor, oxygen. Sulfolobales viruses are highly represented in the metagenome sequence of these Fe(III)-oxide mats [10], which suggests that viral predation of *M*. *yellowstonensis* and other Sulfolobales populations contributes to the turnover of autotrophic biomass and creates reduced carbon sources for heterotrophs.

Genome-enabled stoichiometric modeling is a powerful approach in systems biology for examining metabolic acclimation to environmental stress across size scales from individual cells to communities of interacting populations [20–22]. A summary and graphical representation of stoichiometric modeling can be found in the supplemental information (Figure A in S13 File). Briefly, these approaches construct *in silico* representations of cellular metabolism inferred from genome sequence analysis [23]. The metabolic models define possible routes of electron transport, cellular energy production, carbon acquisition and central metabolism, and include details of the anabolic processes necessary to synthesize biomass. There are two major types of stoichiometric modeling: elementary flux mode analysis (EFMA) and flux balance analysis (FBA). EFMA identifies all distinct and indecomposable routes through a metabolic network; these routes are termed elementary flux modes (EFMs) [24]. EFMs, and non-negative linear combinations thereof, describe all possible physiologies independent of kinetic parameters, which makes EFMA well-suited for evaluating energetic efficiencies of different electron donors, acceptors, and nutrient sources involved in biomass production. FBA identifies optimal routes through a metabolic network that, for example, maximize growth rate for a given substrate uptake rate [25]. The use of optimization to identify these routes and rates for a given set of nutrient uptake rates is ideal for sampling a large metabolic space bounded by known but flexible kinetic parameters. Stoichiometric modeling has been used to predict optimal genotypes in engineered systems [20,26], interpret physiological behavior [21,27], and evaluate the transfer of mass and energy between distinct populations in a natural phototrophic microbial community [22] and others [28].

A primary goal in environmental microbiology is to understand and predict microbial behavior in communities, where interactions among different populations lead to emergent properties, such as enhanced productivity, stability, and robustness [29]. Consequently, the objectives of this study were to 1) construct metabolic network models for major autotroph and heterotroph populations present in oxic zones of high-temperature Fe(III)-oxide mats using metagenome sequence assemblies from representative sites; 2) analyze the use of electron donors and acceptors for the production of biomass and cellular energy under different nutrient limitations; 3) integrate individual population models to examine possible autotroph-heterotroph interactions that may be fundamental to the ecology of microbial communities (e.g. relative population abundances and oxygen competition); and 4) perform a sensitivity analysis based on parameters measured *in situ* to determine model limitations and identify future priorities for field measurements. The approach used here integrates data from the nanoscale of electron transport to microscale oxygen depth-profiles to hot spring-scale measurements of biotic Fe(III)-oxide deposition. Novel insights and governing principles of community structure and function were established through the integration of metagenome-enabled *in silico* approaches and *in situ* measurements.

## Results

### Enzymatic chemolithoautotroph electron donor and acceptor pathways

**Fig 1:**
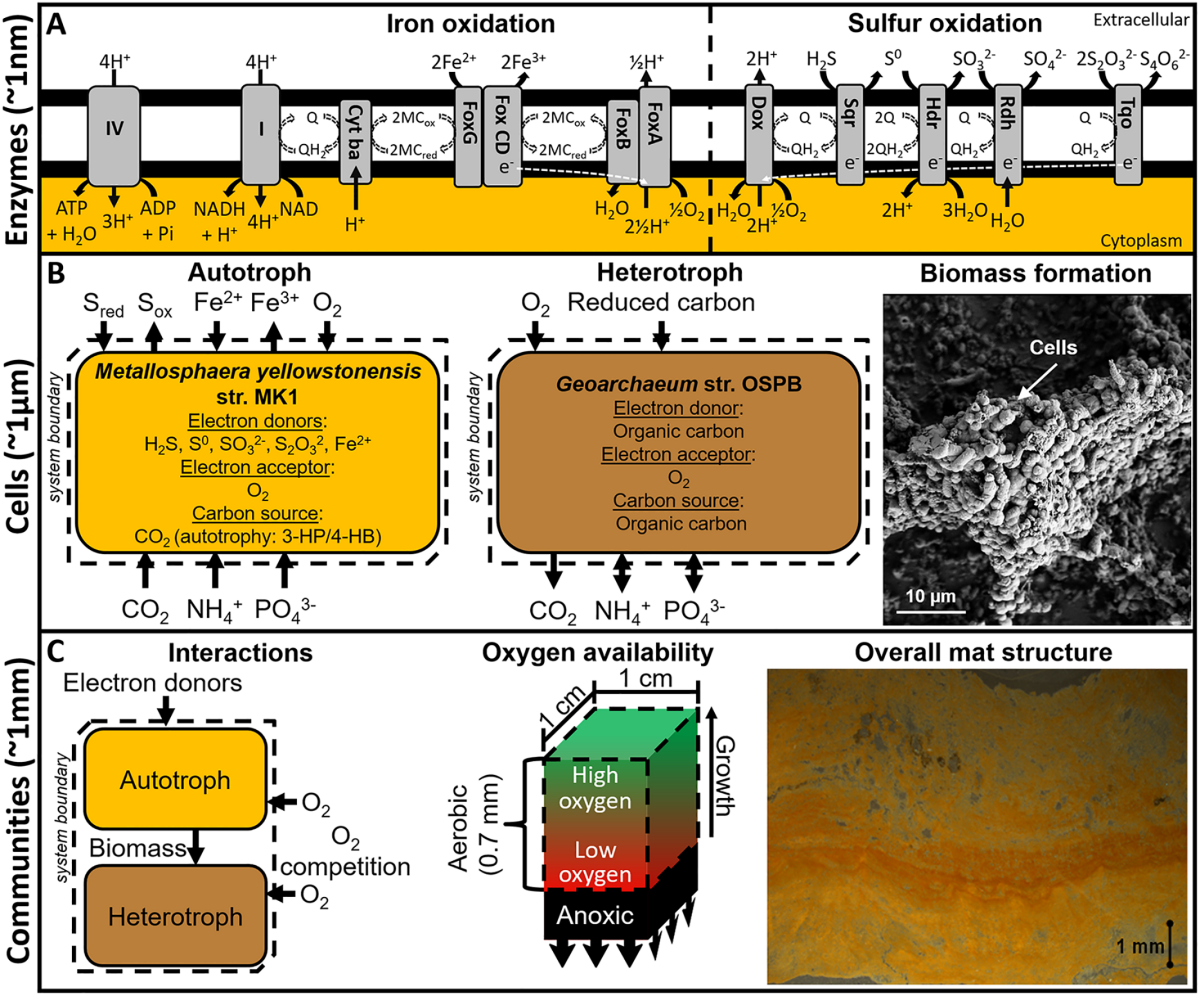
Conceptual representation of the multiscale metabolic interactions between a primary autotroph (*M*. *yellowstonensis*) and heterotroph (*Geoarchaeum* str. OSPB) present in high-temperature (70–80°C) acidic Fe-oxide mats. A) Modeling of these interactions used genome annotations and physiological studies to identify the enzymes for electron donor and acceptor utilization, as depicted here for iron and sulfur oxidation by the primary autotroph. Electron flow is indicated by white dashed lines. B) These enzymes provided the basis for cellular-level models to quantify resource requirements for the primary autotroph and heterotroph to produce biomass and cellular energy from reduced inorganic electron donors and organic carbon, respectively. C) The total resource requirements of each population and *in silico* approaches provided bases to test interaction hypotheses (community-level modeling) using *in situ* measurements, including relative population abundances, carbon isotope fractionation, Fe(III) deposition rates, and oxygen flux into the mat. The system volume considered in these analyses is defined as the aerobic region of the mat (0.07 cm by 1 cm by 1 cm) moving vertically up as the mat grows. The effect of high and low oxygen associated with the top and bottom of the relatively homogeneous aerobic region provided bounds for electron acceptor stresses. Abbreviations: I—energy conserving NADH:ubiquinone oxidoreductase; Cyt ba–cytochrome/quinol oxidase; Fox AB and FoxCDG–Fe(II) oxidation complexes; MCO–Multicopper protein; Q–quinone; QH_2_ –quinol; IV–ATP synthase; Sqr–Sulfur-quinone reductase; Hdr–Heterodisulfide reductase; Rdh–Rhodanese-related sulfur transferase; Tqo–Thiosulfate-quinone oxidoreductase; and Dox–Sulfur associated terminal oxidase.

Genomic data were used to construct *in silico* representations of the electron transport network responsible for the oxidation of Fe(II) and sulfur species and the reduction of oxygen in *M*. *yellowstonensis* str. MK1 (Fig 1). Genomic and physiological evidence indicated that this chemolithoautotroph oxidizes Fe(II) using proteins encoded by the *fox* operon, which are also found in other Sulfolobales [16]. *M*. *yellowstonensis* can also oxidize a variety of sulfur species, including sulfide, elemental sulfur, sulfite, and thiosulfate, to reduce the quinone pool to quinol [16]. Electrons in the quinol pool can drive cellular energy production via oxidative phosphorylation through the reduction of oxygen using a high affinity heme copper oxidase. Alternatively, electrons in the quinol pool can reduce NAD^+^, enter central carbon metabolism, and be used to reduce inorganic carbon via the 3-hydroxypropionate / 4-hydroxybutyrate (3-HP / 4-HB) pathway [17]. These electron transport pathways are hypothesized to provide the majority of energy to the studied microbial communities. The modeled central carbon metabolism of *M*. *yellowstonensis* included the tricarboxylic acid cycle, gluconeogenesis, the pentose phosphate pathway via ribulose monophosphate [30], and the mevalonate pathway [31].

### Cellular resource requirements to produce chemolithoautotroph or organoheterotroph biomass

**Fig 2:**
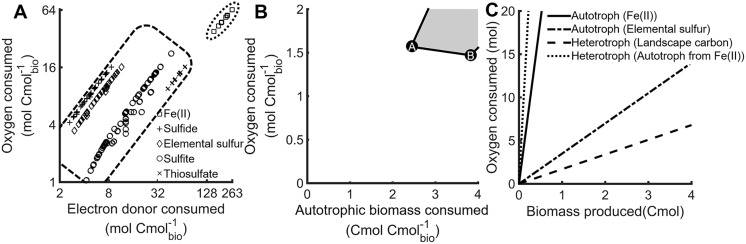
Analysis of electron donor and acceptor resource requirements to produce biomass indicate the autotroph requires more oxygen, and oxygen availability affects carbon requirements of the heterotroph. A) Moles of oxygen and electron donor required to produce one Cmole of *M*. *yellowstonensis* biomass for all biomass producing elementary flux modes, note the log scales. Clusters of electron donors, which include Fe(II), sulfide, sulfur, sulfite, and thiosulfate, are circled by dashed lines. B) Moles of oxygen and autotroph biomass required to produce one Cmole of *Geoarchaeum* str. OSPB biomass. Optimizations for carbon- and oxygen-limited scenarios are marked by points A and B, respectively. C) Relative oxygen consumption plotted as a function of biomass production for autotroph, *M*. *yellowstonensis*, utilizing Fe(II) or elemental sulfur, and for heterotroph, *Geoarchaeum* str. OSPB, utilizing autotroph biomass generated from Fe(II) oxidation or exogenous sources of reduced organic carbon (i.e. landscape carbon).

Electron donor and acceptor pathways for *M*. *yellowstonensis* were integrated into a cellular-level metabolism model to quantify the relationship between growth and the consumption of environmental resources (Fig 1, supplemental material). Biological systems often minimize their requirements for growth limiting resources, providing an ecologically relevant basis for the *in silico* prediction of metabolic phenotypes [32]. A total of 6,337 elementary flux modes (EFMs) were calculated that produce *M*. *yellowstonensis* biomass using the inorganic electron donors, Fe(II), sulfide, elemental sulfur, sulfite, or thiosulfate. Each EFM was plotted as a function of moles of electron donor and moles of electron acceptor required to produce one carbon mole (Cmole) of *M*. *yellowstonensis* biomass (Fig 2A). The moles of electron donor required to form a Cmole of biomass was lowest for sulfide and highest for Fe(II) (Fig 2A), which follows the available free energy predicted for the oxidation of these electron donors coupled to the reduction of oxygen [33]. The oxidation of Fe(II) also required the most moles of oxygen per Cmole of biomass produced of the electron donors evaluated. These relationships were consistent on both a moles of electron donor and moles of oxidized electrons basis (Figure B in S13 File, Table A in S13 File for relevant degrees of reduction).

Genomic analysis of *Geoarchaeum* str. OSPB indicated that this organism is an organoheterotroph with the metabolic potential to utilize a wide variety of reduced carbon species, including biomass macromolecules (i.e. lipids, peptides, polysaccharides, and nucleic acids; see the supplemental material or [34] for a description of reactions involved). Details of *Geoarchaeum* biomass production from components of lysed *Metallosphaera* cells were elucidated in a prior report [34]. Briefly, the production of heterotroph biomass based on the consumption of autotroph biomass as a carbon source was evaluated with respect to the Cmoles of carbon substrate or moles of oxygen required to produce a Cmole of biomass (Table B in S13 File). The production of a Cmole of *Geoarchaeum* str. OSPB biomass required 2.4 Cmoles of autotroph biomass to supply the cellular energy and structural components for growth under carbon-limited conditions (Fig 2B, point A). Production of a Cmole of *Geoarchaeum* str. OSPB biomass under oxygen-limited conditions requires 3.8 Cmoles of autotroph biomass (Fig 2B, point B).

### Cellular oxygen requirements to produce autotroph and heterotroph biomass

The growth of autotroph required substantial inputs of oxygen and was governed largely by the degree of reduction and redox potential of the electron donor (Fig 2C, Table A in S13 File). The predicted growth of autotroph on elemental sulfur required 3.5 moles of oxygen per Cmole of biomass produced while oxidation of Fe(II) required 38 moles oxygen per Cmole of biomass produced. Both predicted oxygen requirements are within the range of measured values for similar organisms growing on sulfur (3.5 moles of oxygen per Cmole biomass, assuming 1.8 * 10^−13^ grams per cell) and iron oxidation (35–104 moles oxygen per Cmole biomass) [35,36].

Different scenarios were used to evaluate heterotroph growth in this system: organic carbon was either made available, opportunistically, as a result of viral predation and subsequent lysis of the autotrophic cells, modeled as free monomer pools, or organic carbon was supplied as the result of a biological strategy where metabolites were excreted from the autotroph. Alternatively, organic carbon was provided from the surrounding landscape (hereafter called landscape carbon) which allowed for heterotrophic growth independent of the examined autotroph. The landscape carbon was modeled to have the same macromolecular composition as autotroph biomass for simplicity (supplemental material). The landscape carbon was produced from electron donors and acceptors external to the system boundaries and therefore did not contribute to oxygen consumption in the mat. Heterotroph growth on landscape carbon required 1.6 moles oxygen per Cmole of biomass produced, which is 54 and 96% less than the oxygen required to support the production of autotroph biomass from either elemental sulfur or Fe(II), respectively (Fig 2C, Table B in S13 File). The lower oxygen requirement for organoheterotrophic growth was due to utilization of reduced carbon substrates as both electron donors and anabolic precursors. By comparison, autotrophic metabolism required substantially more energy to reduce carbon dioxide, which resulted in high oxygen requirements, especially when Fe(II) was the primary electron donor. Consequently, the oxygen requirements to produce heterotroph biomass increased sharply when community-level interactions were included. For example, *Geoarchaeum* str. OSPB requires 2.4 Cmoles of autotroph biomass to produce a Cmole of heterotroph biomass under carbon-limited conditions (Fig 2B, Table B in S13 File). The total, aggregate oxygen requirement to produce heterotroph biomass from the biomass of an Fe(II) oxidizing autotroph included both the oxygen requirements of the heterotroph and the oxygen requirements to produce the autotroph biomass consumed, which resulted in 93 moles of oxygen being consumed per Cmole heterotroph biomass (Fig 2C).

### Community structure based on resource competition

**Fig 3:**
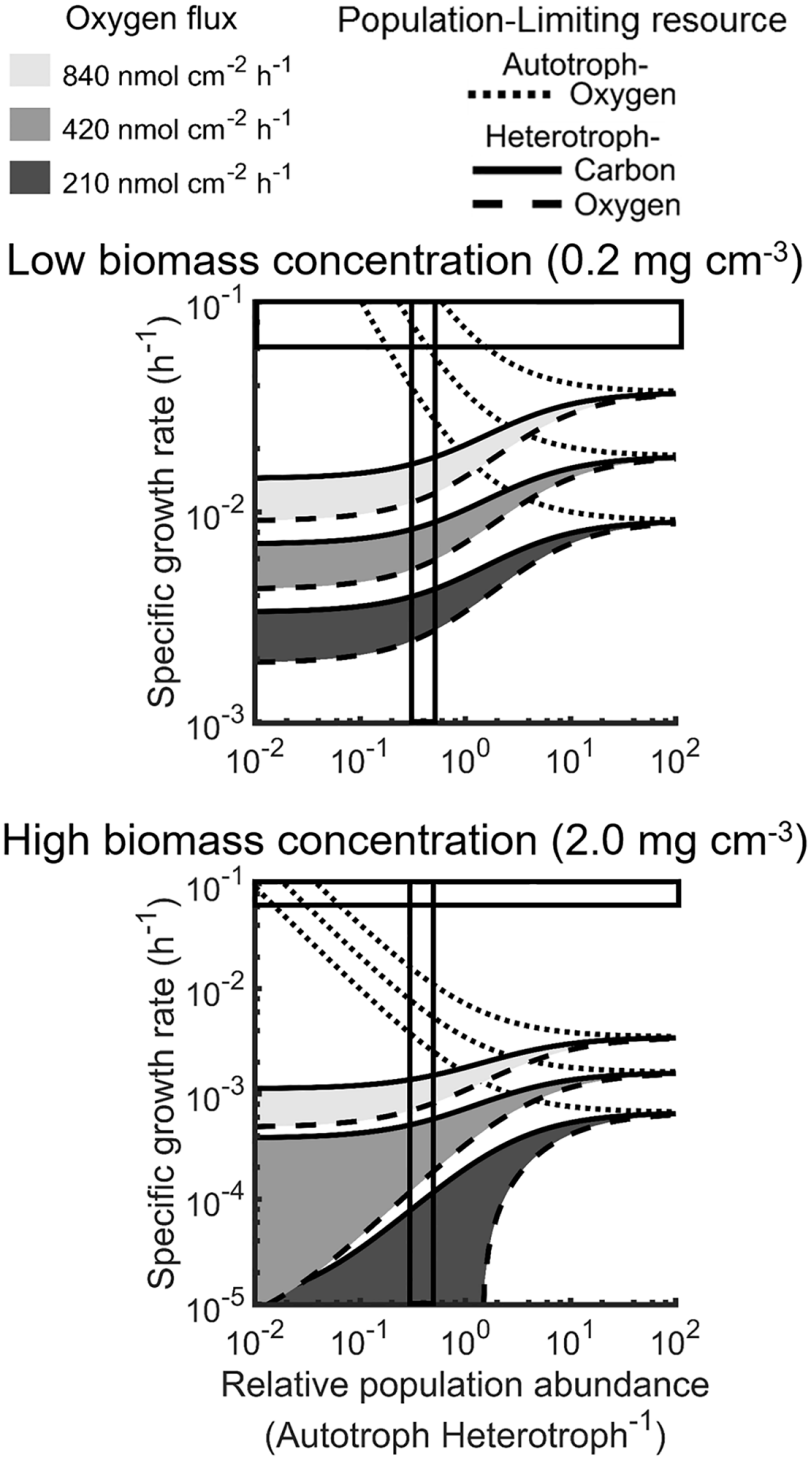
Overlaying simulation of various nutrient limitation, relative population abundance, and *in situ* parameters established a lower limit for the total concentration of biomass in a mat community. Three oxygen fluxes, representing 50, 100, and 200%, of the average observed oxygen flux (420 nmol O_2_ cm^-2^ h^-1^) provided upper and lower bounds of resource availability [12,14]. Each modeled oxygen flux was analyzed using the predicted metabolic strategy for most efficient utilization of oxygen by the autotroph (dotted line) as well as oxygen (solid line) and carbon (dashed line) for the heterotroph. Minimum and maximum active biomass concentrations (top and bottom panels, respectively, note the log scales) and a constant aerobic volume allowed for the prediction of specific growth rates. Consistent with *in situ* sequence data, the predicted relative population abundance was 0.3 to 0.5 autotroph to heterotroph (vertical boxed area) based on maximum *in vitro* specific growth rate (horizontal boxed area) and community stability.

**Fig 4:**
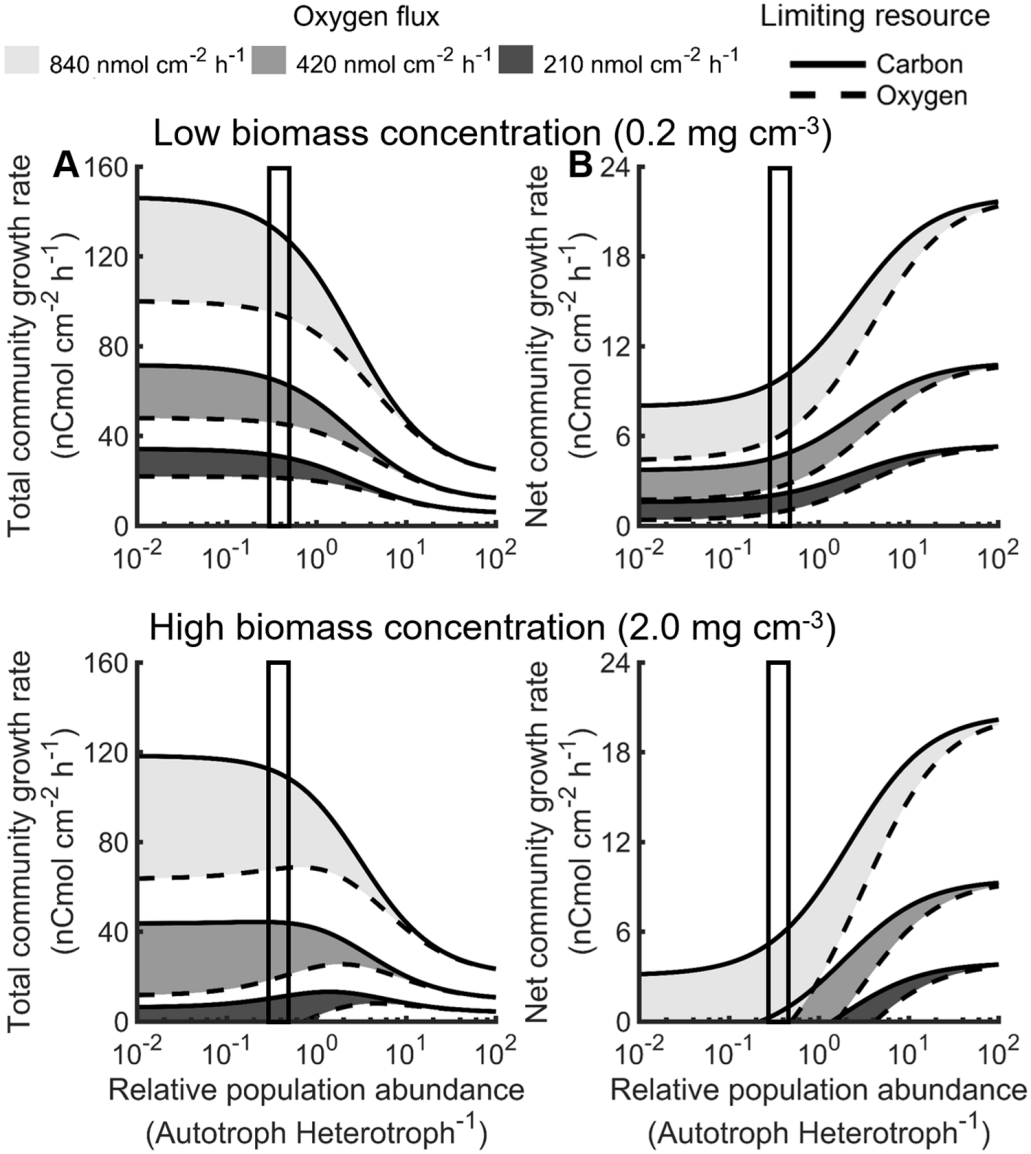
Overlaying simulation of various nutrient limitation, relative population abundance, and *in situ* parameters A) indicates a maximization of total community growth rates and B) established an upper limit for the total concentration of biomass in the studied mat community. Three oxygen fluxes, representing 50, 100, and 200%, of the average observed oxygen flux (420 nmol O_2_ cm^-2^ h^-1^) provided upper and lower bounds of resource availability [12,14]. Each modeled oxygen flux was analyzed using the predicted most efficient metabolic strategy for utilization of oxygen (solid line) and carbon (dashed line). Consistent with *in situ* sequence data, the predicted relative population abundance was 0.3 to 0.5 autotroph to heterotroph (vertical boxed area) based on maximum *in vitro* specific growth rate and community stability.

Oxygen is required to produce biomass and cellular energy for both the autotroph and heterotroph, which results in resource competition between these two trophic levels. Additionally, the heterotroph consumed autotroph biomass as an electron donor, creating a competitive interdependence. Mass balances on the autotroph and heterotroph within the oxic system boundaries (0.7 mm depth, Fig 1C) were described as functions of growth rates, resource requirements, and biomass concentrations (Tables 1 and 2). The system was assumed to be at steady-state for the analyzed time scale of days to weeks, which resulted in a relationship between specific growth rates of the autotroph and heterotroph. This relationship and the flux of oxygen into the system were used to solve for steady-state total community growth rate, which sums both the autotroph and heterotroph, and net community growth rate, which subtracts autotroph carbon consumed by heterotroph from the total community growth rate, (Table 2). The rate of Fe(II) oxidation was then calculated using the autotroph growth rate. These relationships (Table 2) provided a mechanism to examine the effects of oxygen flux and the relative population abundance of autotroph and heterotroph on specific and community growth rates (Figs 3 and 4). Three oxygen fluxes into the mat were analyzed: 50, 100, and 200% of the average *in situ* oxygen flux (420 nmol O_2_ cm^-2^ h^-1^) measured with microelectrodes [12]. *In situ* values of relative population abundances of autotroph to heterotroph (0.3–0.5) were determined based on metagenome analyses from Fe(III)-oxide mat samples collected from Beowulf and OSP springs (Table C and D in S13 File).

**Table 1:** Values and descriptions of parameters used.

Variable	Description	Units	Value
μ_i_	Specific growth rate of population “i”	g g^-1^ h^-1^	Calculated
μ_max_	Maximum growth rate of any population	g g^-1^ h^-1^	0.1
Y_Fe/BA_ / Y_O2/BA_	Iron / oxygen to produce autotroph biomass^a^	mol g^-1^	6.7 / 1.63
Y_Fe/EA_ / Y_O2/EA_	Iron / oxygen to produce autotroph cellular energy	mol mol^-1^	16 / 4
Y_S/BA_ / Y_O2/BA_	Sulfide / oxygen to produce autotroph biomass^a^	mol g^-1^	0.112 / 0.181
Y_S/EA_ / Y_O2/EA_	Sulfide / oxygen to produce autotroph cellular energy	mol mol^-1^	0.25 / 0.5
Y_A/BH_ / Y_O2/BH_	Carbon / oxygen to produce heterotroph biomass^a^	g g^-1^ / mol g^-1^	2.29 (3.45)^b^ / 0.057 (0.054)^b^
Y_A/EH_ / Y_O2/EH_	Carbon / oxygen to produce heterotroph cellular energy	g mol^-1^ / mol mol^-1^	16.8 (8.2)^b^ / 0.35 (0.38)^b^
X_i_	Biomass concentration of population “i”	μg cm^-3^	Calculated
X_Tot_	Biomass concentration of all populations	μg cm^-3^	0.2–2.0
GAM_A_	Cellular energy to sustain autotroph growth	mmol g^-1^	137.6
GAM_H_	Cellular energy to sustain heterotroph growth	mmol g^-1^	120
M_i_	Cellular energy to sustain nongrowing population “i”	mmol g^-1^ h^-1^	0.11
	Depth of oxic zone	cm	0.07
V	Volume of aerobic region	cm^3^	0.07
F	Volumetric flow rate	cm^3^ h^-1^	–
j_O2_	Oxygen flux into the mat	μmol cm^-2^ h^-1^	Calculated
R_Fe_	Iron oxidation rate	μmol cm^-2^ h^-1^	Calculated
	Relative population abundance	g g^-1^	0.3–0.5
	Lowest fraction of biomass carbon from DIC	g g^-1^	0.42 (0.67^d^)^c^
R_Xnet_	Net community growth rate	μmol cm^-2^ h^-1^	Calculated
R_Xtot_	Total community growth rate	μmol cm^-2^ h^-1^	Calculated
	Moles of carbon per gram of autotroph	Cmol g^-1^	0.0430
	Moles of carbon per gram of heterotroph	Cmol g^-1^	0.0438

^a^: Growth associated resource requirements excluding nongrowth resource requirements

^b^: Lowest carbon (oxygen) resource requirements

^c^: Values for Beowulf (OSP) hot springs

^d^: Constrained by mass balance

**Table 2:** Equations used to define mass balances, growth rates, and Fe oxidation rates in terms of other system variables (Table 1).

Parameter	Equation
Autotroph accumulation	(dX_A_/dt) V = μ_A_ X_A_ V–(μ_H_ Y_A/BH_ + M_H_ Y_A/EH_) X_H_ V–F X_A_
Heterotroph accumulation	(dX_H_/dt) V = μ_H_ X_H_ V–F X_H_
Oxygen flux into the mat	j_O2_ = (μ_A_ Y_O2/BAF_ + M_A_ Y_O2/EAF_) X_A_ V + (μ_H_ Y_O2/BH_ + M_H_ Y_O2/EH_) X_H_ V
Total Community Growth Rate	R_Xtot_ = μ_A_ X_A_ V + μ_H_ X_H_ V
Net Community Growth Rate	R_Xnet_ = μ_A_ X_A_ V + μ_H_ X_H_ V–(Y_A/BH_ μ_H_ + M_H_ Y_A/EH_) X_H_ V
Fe oxidation rate	R_Fe_ = (μ_A_ Y_Fe/BA_ + M_A_ Y_Fe/EA_) X_A_ V

The system was analyzed using the oxic zone as system boundaries, which was defined by the *in situ* depth of oxygen penetration, 0.7 mm [14]. The system volume was modeled as homogeneous to simplify the analysis and remained in the oxic zone by moving vertically as Fe(III)-oxide and biomass were deposited.

Simulated total biomass concentrations were bounded by two constraints: 1) maximum specific growth rate of the autotroph and 2) oxygen flux into the mat. A microbial community composed of mostly autotroph was constrained to a minimum biomass concentration of 0.04 mg cm^-3^ by the maximum measured specific growth rate for *M*. *yellowstonensis in vitro* (μ = 0.1 h^-1^ [8,37]) and *in situ* oxygen flux into the mat (420 nmol O_2_ cm^-2^ h^-1^ [12,14]) (Figure C in S13 File). Increasing total biomass concentration decreased the specific growth rate for an autotroph dominated community and increased the feasible steady-state heterotroph abundance in the community at a given oxygen flux into the mat (Fig 3). Two scenarios were examined for heterotrophic growth: 1) a carbon-limited scenario, which was expected to occur at the top of the mat where oxygen is plentiful, and 2) an oxygen-limited scenario, which was expected at the bottom of the oxic zone (Fig 3). Heterotroph acclimation to intermediary oxygen availabilities would be bounded by these two scenarios. The observed *in situ* relative population abundance (0.3–0.5 autotroph per heterotroph) and oxygen flux was predicted to be feasible at steady-state with a total biomass concentration of ~0.2 mg cm^-3^, thereby defining a minimum total biomass concentration for the studied microbial community (Fig 3). Increases in total biomass concentration decreased the specific growth rate for the heterotroph.

The total community growth rate, which was the sum of autotroph and heterotroph produced, was constrained by competition for oxygen and the autotroph biomass requirement of the heterotroph. Increases in flux of oxygen into the mat increased total community growth rate supporting oxygen limitation of the modeled community. Increases in relative population abundance of autotroph to heterotroph decreased the total community growth rate, as it did with the specific rate of the autotroph (Figs 3 and 4A). This trend highlights the available mass and energy in autotroph biomass. This mass and energy can be utilized by heterotrophs with less oxygen required per biomass to further increase the total biomass using the same oxygen flux. Optimized carbon usage by the heterotroph, hypothesized to occur under carbon limitation, increased community growth rates by minimizing the autotroph biomass required to produce heterotroph. Conversely, optimized oxygen usage by the heterotroph increased carbon requirements for the heterotroph and decreased all steady-state growth rates by consuming more autotroph biomass (Figs 3 and 4).

The net community growth rate, which quantified autotroph and heterotroph accumulation as opposed to production, was constrained by competition for oxygen and the heterotrophic requirement for autotroph biomass. The net and total specific rate of an autotroph only community was equal because no biomass was consumed (Fig 4). Net community growth rate increased with oxygen flux, as observed for specific rate, and relative population abundance of autotroph to heterotroph (Fig 4B). The maximum net community growth rate occurs in a community composed of mostly autotroph and is equal to the total growth rate of the autotroph. The observed oxygen flux into the mat (420 nmol O_2_ cm^-2^ h^-1^ [12,14]) and biomass concentrations above 0.7 mg cm^-3^ resulted in negative net community growth rates, which are not sustainable in a steady-state community, due to the minimum metabolic activity to maintain an active population (Figure C in S13 File). The observed relative population abundances and oxygen flux predicted a maximum feasible, total biomass concentration of ~ 2.0 mg cm^-3^, above which the net community growth rate was negative. A negative net community growth rate indicated that the total biomass concentration in the mat was not sustainable and would decrease for the oxygen fluxes simulated (Fig 4B).

### Community electron donor sensitivity analysis using multiscale, hybrid EFMA+FBA models

**Fig 5:**
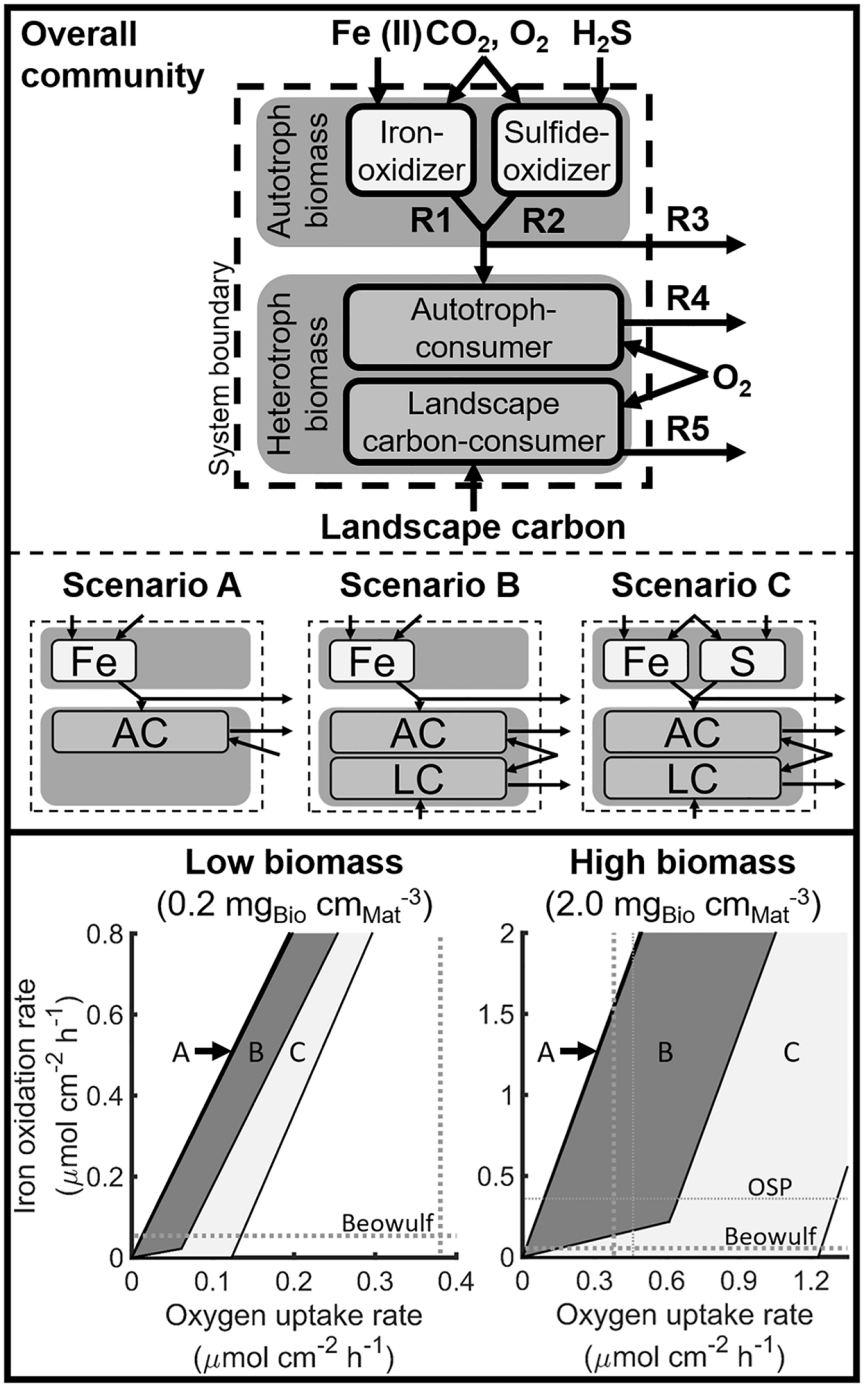
Sensitivity analysis of system behavior as a function of geochemical variation provides physiological context for *in situ* measurements. Three scenarios (middle panel) predicted the metabolic space feasible for autotroph-heterotroph interactions of the entire community (top panel). Scenario A analyzes a community composed solely of an Fe(II) oxidizing autotroph (represented by R1 or Fe) and autotroph-consuming heterotroph (R4 or AC), which are present at relative population abundances of 0.3 and 0.5 autotroph to heterotroph. Scenario B analyzes the impact of additional carbon sources for the heterotroph (R5), such as the consumption of landscape carbon (LC), while enforcing 42 to 99% of total biomass carbon was autotroph in origin based on the *in situ* relative system carbon isotope signature. The decrease in slope toward the bottom of scenario B (lower panel) is due to mathematical resolution and the required carbon fractionation in biomass. Scenario C determines the impact of an additional electron donor (sulfide) for the autotroph (R2 or S) in addition to a landscape carbon source for the heterotroph. The net production of autotroph is denoted by R3. Maximum specific growth rate and system volume were set to 0.1 h^-1^ and 0.07 cm^-3^, respectively. Biomass and cellular energy yields were determined by elementary flux mode analysis, which were then used as inputs to flux balance analysis to predict rates. Averages of observed Fe(III)-oxide deposition (0.054 and 0.36 μmol cm^-3^ h^-1^ for Beowulf and OSP, respectively) and oxygen uptake rates (0.38 and 0.46 μmol cm^-3^ h^-1^ for Beowulf and OSP, respectively) [12,14] (dotted lines) are shown for comparison.

*In situ* measurements in natural ecosystems are complicated by numerous variables, such as seasonal variation in weather, wind, precipitation, temperature, and inherent heterogeneity. A sensitivity analysis was performed using sulfide and landscape carbon as alternative electron donors for the autotroph and heterotroph, respectively, using a multiscale, hybrid EFMA and FBA approach. The hybrid methodology first identified cellular-level, metabolic acclimation strategies to simulated environments, such as carbon- or oxygen-limited environments, using EFMA. The analysis hypothesized that organisms maximized the desired product, such as biomass or cellular energy, per limiting resource utilized (discussed above) (Fig 2). The same optima are calculable using FBA, but cellular-level EFMA provides additional insights, such as the number of reactions and distribution of suboptimal yields, which are difficult or prohibitive to determine using FBA [34]. The EFM resource requirements to produce biomass and associated maintenance energy were adjusted for specific growth rate using the relationship discussed in Pirt *et al* [38]. These cellular-level activities were represented by overall resource transformation reactions, quantified using the model exchange reactions, then incorporated into a model of community-level function, which was analyzed using FBA. FBA provides a convenient tool for assessing the impact of rate constraints and different modeling optimization criteria, such as maximizing net or total community growth rate, on the feasible range of metabolic activities. The hybrid FBA simulations evaluated three different electron donor scenarios (A, B, and C in Fig 5, Figure D in S13 File).

Firstly, Fe(II) and autotroph biomass were the only electron donors for the autotroph and heterotroph, respectively (Scenario A), which addressed the capacity of the community to oxidize Fe(II) based on varying oxygen flux into the mat (Fig 5, Figure E in S13 File). The resource requirements for the heterotroph to produce biomass and cellular energy were bounded by results from the carbon- and oxygen-limited EFMA simulations to approximate the high and low oxygen regions in the mat, respectively. The growth rate constraints used for FBA are described in Figure D in S13 File and Table 1 and included relative population abundance of autotroph to heterotroph, total biomass concentration, and the maximum specific growth rates of autotroph and heterotroph. The maximum specific growth rates of both populations were set to 0.1 h^-1^ based on *in vitro* studies of *M*. *yellowstonensis* [8,37]. Maximization of net and total community growth rate impacted the simulated rates but not the range of metabolic activities feasible by the system.

Secondly, autotroph biomass and landscape carbon were both evaluated as possible carbon sources for the heterotroph (Scenario B). This scenario examined the effect of 42 to 99% of the biomass carbon in the system having been supplied by the Fe(II)-oxidizing autotroph with the balance from exogenous, landscape carbon (Fig 5, Figure F in S13 File) as determined by *in situ* isotopic analyses [17]. The biomass carbon origins were simulated by setting the relative population abundance of autotrophy-based populations (i.e. autotroph and autotroph consuming heterotroph) and the landscape carbon consuming heterotroph. Growth of the autotroph-consuming heterotroph resulted in a lower limit for iron oxidation below which sustaining the heterotrophic population would consume more autotroph than produced (Fig 5, Figure F in S13 File).

Finally, sulfide was evaluated as an alternative electron donor for the autotroph (Scenario C). Elemental sulfur and sulfide can be present at low levels in Fe(II) oxidizing mats. The presented analysis only considered sulfide as it was predicted to have the largest impact (Fig 2A, Figure G in S13 File). Autotrophy was simulated to vary between oxidation of sulfide exclusively or Fe(II) exclusively with a total autotroph maximum specific growth rate of 0.1 h^-1^; therefore, as Fe(II) oxidation rates increased, less oxygen could be directed toward sulfide oxidation (Fig 5, Figure G in S13 File). A scenario where heterotroph was able to consume Fe(II) and/or sulfide oxidizing autotroph but not landscape carbon, predicted a trend similar to scenario C.

The three scenarios were compared to the *in situ* measurements of Fe(III)-oxide deposition and oxygen flux into the mat. The average *in situ* measurements for OSP Spring were within the predicted metabolic space for both scenarios B and C in a microbial mat that has a high biomass concentration (~ 2.0 mg cm^-3^ as determined above). This overlap between *in silico* and *in situ* observation suggests that oxidation of landscape carbon or combinations of landscape carbon and reduced sulfur species could account for the additional aerobic activity in OSP Spring communities. The average *in situ* measurements for Beowulf spring were within the predicted metabolic space for scenario C for the high biomass concentration simulations, which indicated that the community would have to use landscape carbon, as well as Fe(II) and sulfide oxidation to account for the oxygen consumption. At lower biomass concentrations the metabolic space covered by each scenario decreased, excluding the *in situ* measurements of both Beowulf and OSP Springs, which indicated if these springs have such low biomass concentrations there must be additional oxygen consumption occurring, such as the oxidation of additional electron donors (see Discussion).

## Discussion

Microbial processes span multiple spatial scales from metabolites, enzymes, individual cells, populations, and communities to microbial mats that can ultimately impact planetary biogeochemical cycling (Fig 1). Metagenomic data from Fe(III)-oxide mats in YNP provided a foundation for identifying enzymes responsible for electron transport in a dominant autotroph and heterotroph that occur within the oxic zones of Fe(III)-oxide microbial mats. Biochemical pathways for autotrophic and heterotrophic metabolism were integrated into stoichiometric models to quantify cellular-level resource requirements for biomass and cellular energy production (Fig 2). *In situ* measurements of relative population abundances (i.e. metagenomes) and fractions of autotrophic- versus landscape-based biomass carbon (i.e. stable carbon isotopes [17]) provided context for modeling microbial interactions within these communities. In addition, *in situ* measurements of oxygen flux into these mats using microelectrodes [12,14], and Fe(III)-oxide accretion rates from long-term temporal studies [12] (Fig 5) were integrated with the *in silico* models to quantify possible interactions between a dominant primary producer and secondary consumer, as well as the total contribution of these populations to the biogeochemical activity of the natural Fe(III)-oxide mats. Autotroph-heterotroph interaction modeling indicated that at least 98% of the measured oxygen flux *in situ* is consumed by the autotroph (e.g. *M*. *yellowstonensis*). This is due in part to the relatively low energy content of the electron donor (i.e. Fe(II)) and the high energy demands of carbon dioxide fixation. Conversely, heterotrophic activity had little effect on predicted Fe(II) oxidation rates as a function of oxygen consumed (Scenario A and B in Fig 5), though it represented the major fraction of steady-state biomass. The relatively high energy content of autotroph biomass compared to Fe(II) meant heterotroph growth increased the total biomass produced at the cost of a decreased net community growth (Fig 4).

The turnover of autotroph biomass is important to nutrient and energy flux through the mat community. It has been hypothesized that viral predation and subsequent cell-lysis represents a major mechanism of carbon cycling in microbial communities [39,40]. Numerous Sulfolobales viruses have been identified [41,42], and more specifically, the genome of *M*. *yellowstonensis* contains an extremely high abundance of transposases [16] and viral CRISPR spacers, which suggests that viruses are important in the life-cycle of these organisms. Assembled sequences of Sulfolobales viruses have been obtained from Fe(III)-oxide microbial mats [10], and the lysis of archaeal cells by viruses has been observed *in situ* using scanning electron microscopy (Inskeep, unpl). The turnover of autotroph biomass due to viral predation releases organic carbon enabling the production of heterotroph biomass, which leads to increased total community activity (Fig 4A). The predicted autotroph turnover is greater than 80% at the observed relative population abundances (Figure H in S13 File). To put this turnover value in perspective, it falls within the experimentally observed range of 26 to 1200% reported in other studied geothermal systems [43]. Cell lysis provides one mechanism of carbon and energy exchange; alternatives, such as metabolite exchange, could also contribute to these relative rates and abundances. Metabolite exchange is not expected to be the sole mechanism of exchange in these communities as it would necessitate greater than 80% of all autotrophically fixed carbon be secreted (Figure H in S13 File). The presented analysis does not exclude these mechanisms; indeed, a combination of lysis and metabolite exchange likely occur to varying degrees in all microbial communities. In addition to the lysis mechanism of carbon and energy transfer, simulations were run which considered only metabolite exchange between autotroph and heterotroph populations subject to the previously described community constraints. The metabolite exchange mechanism was simulated where the autotroph secreted monomer distributions consistent with the autotroph biomass composition to facilitate comparison with simulations of the lysis mechanism. The predicted maximum and minimum biomass concentrations were relatively unaffected (supplemental material). The simulated lysis mechanism required cellular energy expenditures by the autotroph to polymerize the monomer pools; this cellular energy was lost to the community when the organic material was transferred to the heterotroph. This energy expenditure was not required during simulated metabolite exchange mechanism conserving community energy and the oxygen used to produce it. However, the oxygen required for polymerizing monomer pools was very small relative to the oxygen required to fix DIC resulting in minimal differences in the predicted maximum and minimum biomass concentrations (Supplemental material).

The relative population abundances observed *in situ* suggest that the Fe(III)-oxide mat communities have metabolic activity that maximize energy acquisition from the environment thereby making them more competitive than those that exhibit lower rates of energy acquisition; this theory is known as the maximum power principle [44]. The ecological benefit to maximizing energy acquisition may be the dilution of autotroph biomass, which increases community diversity and has been shown to promote resilience and stability of the entire community from phage and/or predatory bacteria [45,46]. Applying the maximum power principle to metagenome-derived models may provide theoretical context for community structure-function relationships in other systems.

Analysis of the carrying capacity (i.e. maximum sustainable population numbers) of Fe(III)-oxide mats indicated that minimum and maximum total biomass concentrations exist where the specific growth rate of autotroph or oxygen flux into the mat become limiting, respectively. The minimum predicted biomass carrying capacity of 0.2 mg cm^-3^ (Fig 3) corresponds remarkably well with the calculated biomass concentration in Beowulf Spring (0.3 mg cm^-3^) (Table E in S13 File), which suggests that this microbial community is governed by the maximum specific growth rate of autotroph. Increases in heterotroph abundance would require an autotroph specific growth rate higher than the value observed *in vitro* [8]. This reduction in biomass concentration, and therefore autotroph limitation, is supported by high channel flow rate (20–30 cm s^-1^) and associated shear force in Beowulf Spring [12]. Conversely, the maximum predicted biomass carrying capacity of 2.0 mg cm^-3^ (Fig 4B and Figure I in S13 File) corresponds well with the calculated biomass concentration of OSP Spring (2.2 mg cm^-3^) (Table E in S13 File), which suggests that biomass production at this site is governed by the flux of oxygen. Indeed, in OSP Spring the channel flow rate is 10-fold lower than Beowulf (2–5 cm s^-1^), which results in less shear force and more limited oxygen transfer [12]. The presented modeling framework combined with ecological theory provides a powerful tool for understanding factors that control community structure and function, and can be applied to many natural and/or engineered microbial systems.

A variety of additional oxygen consuming processes in the Fe(III)-oxide mats could explain the higher amounts of measured oxygen flux relative to measured Fe(III)-oxide deposition rates [14] (Fig 5). For example, at least three major oxygen consuming processes (i.e., additional electron donors) could account for the higher measured oxygen flux than predicted for Fe(II) oxidation alone; these include the oxidation of reduced sulfur species, As(III), and reduced carbon from landscape carbon sources [14]. Small amounts of sulfide (< 5 μM) and occasional flocs of elemental sulfur can be present in Fe(III)-oxide depositing zones of acid-sulfate-chloride springs, such as Beowulf Spring, where high sulfide at discharge results in the deposition of elemental sulfur upstream of Fe(II) oxidation [5]. However, analysis of landscape carbon as an additional stimulant for heterotrophic activity could not account for the Fe(III)-oxide deposited for a given oxygen flux in the low biomass concentration simulations expected to be more representative of Beowulf Spring (Fig 5). Additionally, 25 to 30 μM As(III) is present in many acid-sulfate-chloride springs, and has been shown to be oxidized to As(V) by a bacterial autotroph population(i.e. *Hydrogenobaculum spp*.), which is commonly found in high abundance upstream of the examined mat community [47]. Elemental analysis of the poorly-crystalline Fe(III)-oxide phases indicates that significant amounts of As(V) are incorporated into the solid phase [5]. Any of these reduced species would result in consumption of additional oxygen if oxidized and warrant further study.

The integration of *in situ* measurements and metagenome-based *in silico* analyses provide unprecedented approaches to understand microbial interactions and community function. The analyses applied in the presented study provide context for the relative population abundance of autotrophs and heterotrophs, the minimum and maximum biomass concentration of microbial mats, and the possible effects of additional electron donors. The multiscale analyses presented here suggests that microbial interactions contribute to emergent properties of complex organization, such as increased productivity. These principles are likely shared across microbial and macro ecology.

## Materials and methods

### Genome analysis and individual model construction

*In silico* stoichiometric models were constructed for autotroph *M*. *yellowstonensis* str. MK1 (NCBI Taxon ID 671065, GOLD ID Gi04920) and heterotroph *Geoarchaeum* str. OSPB (NCBI Taxon ID 1448933, GOLD ID Gi0000638) in the following 5 step process. 1) Initial models were constructed using RAST [48–50]. 2) The elementally and electronically balanced reactions that represent carbon and energy metabolism were manually curated based on genome sequence (Joint Genome Institute—Integrated Microbial Genomes (JGI-IMG) [51]), prior genome analyses [6–8,10,16,17], literature surveys, and the MetaCyc [52,53] and KEGG databases [54]. 3) Reactions were assumed to close pathway gaps that would have resulted in auxotrophy or deviated from observed physiological behavior [17]. 4) The modeled macromolecular compositions of biomass for each population were 2% DNA, 11% lipid, 11% polysaccharide, 16% RNA, and 60% protein based on previous reports [55]. Genomes of each organism were mined for monomer distributions of DNA, RNA, and protein, based on the GC content, the ribosomal subunits, and the average amino acid distribution of all protein encoding genes, respectively (supplemental material, [51]). 5) Maintenance energy requirements were adjusted to calibrate the *M*. *yellowstonensis* and *Geoarchaeum* str. OSPB models to observed yields for *Acidothiobacillus ferroxidans*, a representative thermoacidophilic autotrophic Fe(II) oxidizing bacterium [56,57] and *Alicyclobacillus acidocaldarius*, a representative thermoacidophilic heterotrophic bacterium [58], respectively. Nongrowth associated maintenance energy (0.11 mmol ATP g biomass^-1^ h^-1^ for both *M*. *yellowstonensis* and *Geoarchaeum* str. OSPB) was calculated for 65°C assuming 50 KJ mol ATP^-1^ (supplemental material) [59], which determined the growth associated maintenance energy (171 and 149 mmol ATP g biomass^-1^ for *M*. *yellowstonensis* and *Geoarchaeum* str. OSPB, respectively).

EFMA-based cellular metabolisms (Fig 2A and 2B) were used as input reactions for FBA-based sensitivity analysis of electron donors available to the community. Briefly, the net exchange reactions from the EFMs were expressed on a specific biomass or specific cellular energy basis, such as iron per autotroph (mol Fe(II) per gram of autotroph), by normalizing to the biomass formed. The maximum specific growth rate (0.1 h^-1^), relative population abundances (0.3–0.5 autotroph to heterotroph), and DIC incorporation fractionation were then used to establish constraints on the maximum and minimum rates and population abundances (See supplemental material for explicit constraints and optimization criteria for the different environmental electron donor scenarios).

### Computational packages and analyses

*In silico* stoichiometric models were constructed using CellNetAnalyzer version 2014.1 [60], and exported to RegEFMTool version 2.0 [61] to enumerate EFMs for each metabolic model. The macronutrients available to both populations were modeled as illustrated in Fig 1. Metabolic reconstructions for both populations, including reactions, genomic evidence, specific literature references, metabolites, stoichiometric balance, and gene regulatory rules can be found in the supplemental material. SBML files provided were exported and tested for import using CellNetAnalyzer version 2017.3 and the CNA SBML parser. Community FBA was performed using functions from the COBRA Toolbox [62] and overall reactions obtained from EFMs deemed ecologically competitive based on resource utilization. Functions used for the community analysis are available in the supplemental material. All computations were processed on a machine with at most two Intel Xeon X5690 and 120 GB RAM.

## Supporting information

S1 File AutoHeteroBiomass. (M)

S2 File Auto_Hetero_Biomass_Exchange. (M)

S3 File BioMatAnalyzer. (M)

S4 File DRAPuller. (M)

S5 File Fig 2A. (M)

S6 File Fig 2C. (M)

S7 File Figurepuller. (M)

S8 File Geo. (XML)

S9 File Geo_Reaction_Workbook. (XLSX)

S10 File Myell. (XML)

S11 File Myell_Reaction_Workbook. (MAT)

S12 File Myell_output. (XLSX)

S13 File SupplementalMaterial. (DOCX)
