# Supplementary material for: Multiscale analysis of autotroph-heterotroph interactions in a high-temperature microbial community
Source: PLoS Comput Biol. 2018 Sep 27;14(9):e1006431. doi: 10.1371/journal.pcbi.1006431 (PMC6177205; doi:10.1371/journal.pcbi.1006431)
Supplement: S13 File — (DOCX) [file pcbi.1006431.s013.docx]

**Supplemental material for “Multiscale analysis of autotroph-heterotroph interactions in a high-temperature microbial community”**

Kristopher A. Hunt, Ryan deM. Jennings, William P. Inskeep, and Ross P. Carlson

**Supplemental Material Table of Contents**

Page # Section

2 Basic stoichiometric modeling concepts

3 EFMA of individual population models

3 Heterotroph turnover and electron acceptor recycling

3 Approximation of population independent oxidation of reduced sulfur species

4 Resource requirements for autotroph and heterotroph biomass production

6 Determination of relative population abundance of autotroph to heterotroph

7 Additional limits for total biomass concentrations in the mat community

8 Graphical representations of metabolic space simulated using hybrid EFM-FBA

12 Autotroph biomass turnover to sustain the heterotrophic population

13 Sensitivity analysis of nongrowth associated maintenance energy

14 Determination of total biomass concentration from *in situ* Fe measurements

15 Impact of metabolite exchange on the maximum and minimum biomass concentration

16 References

**Additional supplemental files**

*M. yellowstonensis* model

Excel (Myell_Reaction_Workbook.xlsx) and SBML (Myell.xml)

*Geoarchaeum* model

Excel (Geo_Reaction_Workbook.xlsx) and SBML (Geo.xml)

MATLAB code for analyses

AutoHeteroBiomass: Analysis of the impact of relative abundance and oxygen flux on biomass production in an iron oxidizing autotroph heterotroph community, Figures 3 and 4.

AutoHeteroBiomassExchange: Analysis of the impact of relative abundance and oxygen flux on biomass production in an iron oxidizing autotroph heterotroph community based on metabolite exchange, Figures 3 and 4

BioMatAnalyzer: Analysis of the metabolically feasible space achievable with varying electron donors and carbon sources. This is also the code that performs the FBA portion of the community analysis.

DRAPuller: Used by BioMatAnalyzer to calculate the FBA-based flux vectors for the space being examined.

Figurepuller: Used to plot the results of BioMatAnalyzer, Figure 5.

Figure2A: Used to analyze the Myell_output and generate figure 2A.

Figure2C: Code that generates figure 2C.

Myell_output: EFMA output for *Metallosphaera yellowstonensis* MK1.


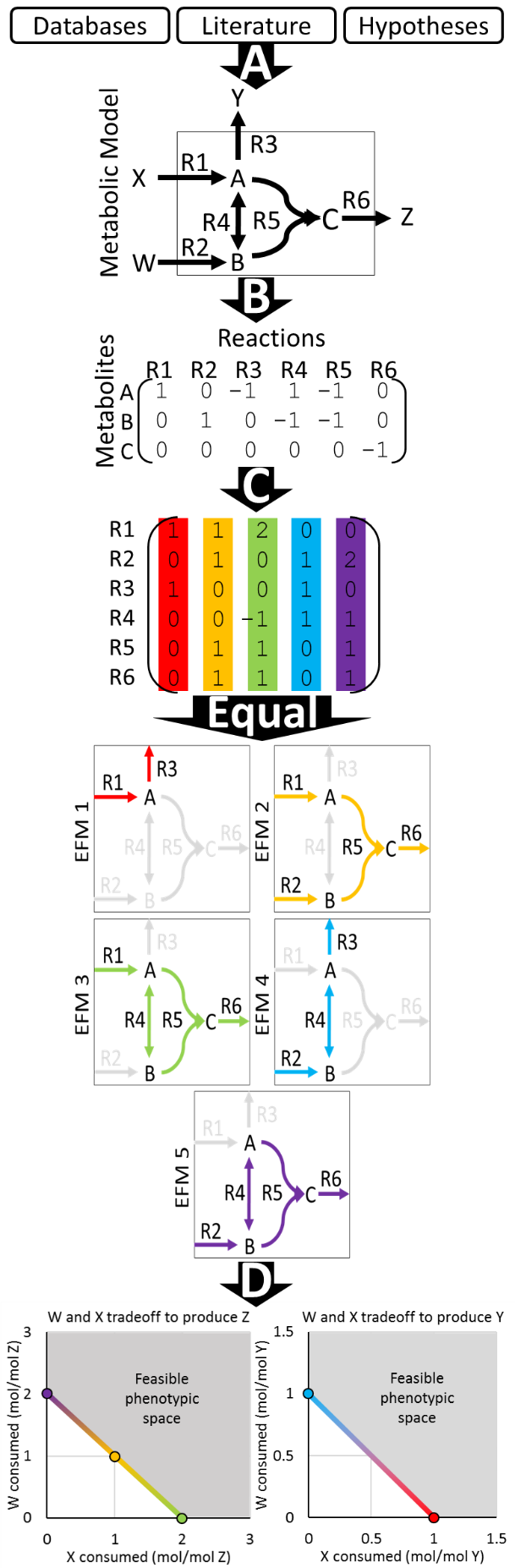


Basic stoichiometric modeling concepts

**Figure A**. Summary of metabolic model building, stoichiometric analysis, and application of ecological theory. A) The metabolic model is a compilation of genomic annotations from databases, experimental yields and efficiencies reported in the literature, and hypothesized reactions. Hypothesized reactions include those missing from annotations, but expected due to literature data (*i.e*. an organism that grows on a mineral medium has complete biosynthetic pathways). B) The model is represented as a series of reaction equations correlating the consumed metabolites and the produced metabolites (i.e. R_i,_ where i corresponds to the reaction number and is written in terms of metabolites A, B and C; W, X, Y, and Z are sources and sinks). The reaction equations can be written concisely as a mathematical matrix, positive coefficients are reaction products and negative coefficients are reaction substrates. C) These reaction equations are analyzed to identify metabolic routes through the network that are stoichiometrically balanced (i.e. all metabolites produced must be consumed and *vice versa*) and all reactions are limited to directions permitted by chemical thermodynamics. In flux balance analysis (FBA), these routes must also satisfy an objective function (e.g. maximize flux through R6 while constraining the magnitude of flux through reactions R1 and R2). Elementary flux mode (EFM) analysis finds all the simplest, genetically distinct routes through the network (i.e. the five possible EFMs (color coded) for the sample metabolic model). D) Application of ecological theory to the analysis of these routes predicts competitive metabolic behavior. The theory assumes the reactions used to produce the desired product (e.g. biomass or cellular energy) will minimize the resource cost for the limiting nutrient (e.g. carbon, oxygen, or cellular volume). In the context of the depicted example, if W is limiting and a population must produce Z, theory states the optimal strategy would be along the x-axis (plot to the left). Conversely, limitation of X to produce Z would be along the y-axis (plot to the left). In addition, alternative products may favor alternative metabolic strategies (e.g. production of Y in the plot to the right). Additional review articles on stoichiometric modeling: [1–4]. Reproduced from [5].

EFMA of individual population models

The RegEFMTool software package employs user-defined gene regulatory rules governing the co-occurrence of reactions within an EFM to simplify the model, thus decreasing the computational burden. The incorporation of gene regulatory rules removed futile cycles that both synthesized and degraded potential carbon or energy sources (*Geoarchaeum*) or used multiple electron donors (*M. yellowstonensis*). The cellular-level EFMA of Geoarchaeum can be found in Hunt et al 2016. The cellular-level EFMA of *M. yellowstonensis* simulated a single carbon source (carbon dioxide) and EFMs were enumerated in full through a single simulation. The resulting output was processed using the provide supplemental file “Figure 2A”.

Heterotroph turnover and electron acceptor recycling

Little is known regarding viruses specific to *Geoarchaeum*. It is highly likely that *M. yellowstonensis* is subject to significant viral predation as well. Cyclical catabolism of heterotrophic biomass by heterotrophic organisms is highly probable but for every new Cmole of heterotroph, at least 2.4 Cmoles of old heterotroph would be consumed. In anoxic environments, a Cmole of anaerobe biomass is expected to require at least 10 Cmoles of carbon source. This indicates the recycling of heterotroph biomass would decrease by 2- to 10-fold with each cycle. Additionally, assuming all autotroph biomass is consumed by anaerobic heterotrophs, for every mole of Fe(II) or sulfide oxidized by the autotroph at most 0.03 or 0.21 moles, respectively, would be reduced by the anaerobe. However, the carbon consumed by anaerobes is expected to be closer to 10 % of the total produced, further reducing the impact of anoxic electron cycling in these mats.

Approximation of population independent oxidation of reduced sulfur species

Populations of *M. yellowstonensis* have not been found in sulfidic systems of acid-sulfate-chloride geothermal springs of NGB, despite the fact that these Sulfolobales contain the genetic potential for the oxidation of elemental sulfur and have been shown to do so in culture in the absence of significant sulfide [6]. Iron oxidation genes (i.e. *fox* complex) are more highly expressed under field conditions than sulfur or organic carbon-associated oxidases [7], which is consistent with the observed localization of *M. yellowstonensis* in zones with higher oxygen concentrations (e.g., DO > 20 µM). Elemental sulfur generally co-occurs with high sulfide concentrations (e.g., DS > 10 µM) in acid-sulfate and/or acid-sulfate-chloride geothermal springs of YNP, which precludes the necessary oxygen flux to support the generation of *M. yellowstonensis* biomass.

Resource requirements for autotroph and heterotroph biomass production

**Table A.** Electron donors and acceptor required to produce biomass for the most efficient metabolisms identified through elementary flux mode analysis for the autotroph, *M. yellowstonensis*.

| Electron Donor | Electron Donor per Biomass^a^ | Oxygen per Biomass^b^ | Degree of Reduction |
| --- | --- | --- | --- |
| Fe(II) | 156 | 38.0 | 1^c^ |
| Sulfide | 2.6 | 4.2 | 8 |
| Elemental sulfur | 3.0 | 3.5 | 6 |
| Sulfite | 4.2 | 1.0 | 2 |
| Thiosulfate | 42.3 | 9.5 | ^d^ |

^a^: Moles of electron donor consumed per Cmole of biomass produced

^b^: Mole of oxygen consumed per Cmole of biomass produced

^c^: Assuming Fe(III) has a degree of reduction of 0

^d^: One electron is released per thiosulfate oxidized

**Table B.** Carbon sources and electron acceptor required to produce biomass for the most efficient metabolisms identified through elementary flux mode analysis for the heterotroph, *Geoarchaeum*.

|  | Carbon-limitation | |  | Oxygen-limitation | |  |
| --- | --- | --- | --- | --- | --- | --- |
| Carbon Source | Carbon per Biomass^a^ | Oxygen per Biomass^b^ |  | Carbon per Biomass^a^ | Oxygen per Biomass^b^ | Degree of Reduction^c^ |
| Aggregate Biomass | 2.4 | 1.6 |  | 3.8 | 1.5 | 4.3 |
| Lipid | 2.1 | 1.9 |  | 2.9 | 1.9 | 5.9 |
| Peptide | 2.6 | 1.7 |  | 3.9 | 1.6 | 5.0 |
| Polysaccharide | 2.6 | 1.5 |  | 4.6 | 1.2 | 4.0 |
| RNA | 3.1 | 1.4 |  | 5.4 | 1.1 | 4.1 |

^a^: Cmoles of carbon substrate consumed per Cmole of biomass produced

^b^: Mole of oxygen consumed per Cmole of biomass produced

^c^: Degree of reductions were calculated on NH_3_ bases


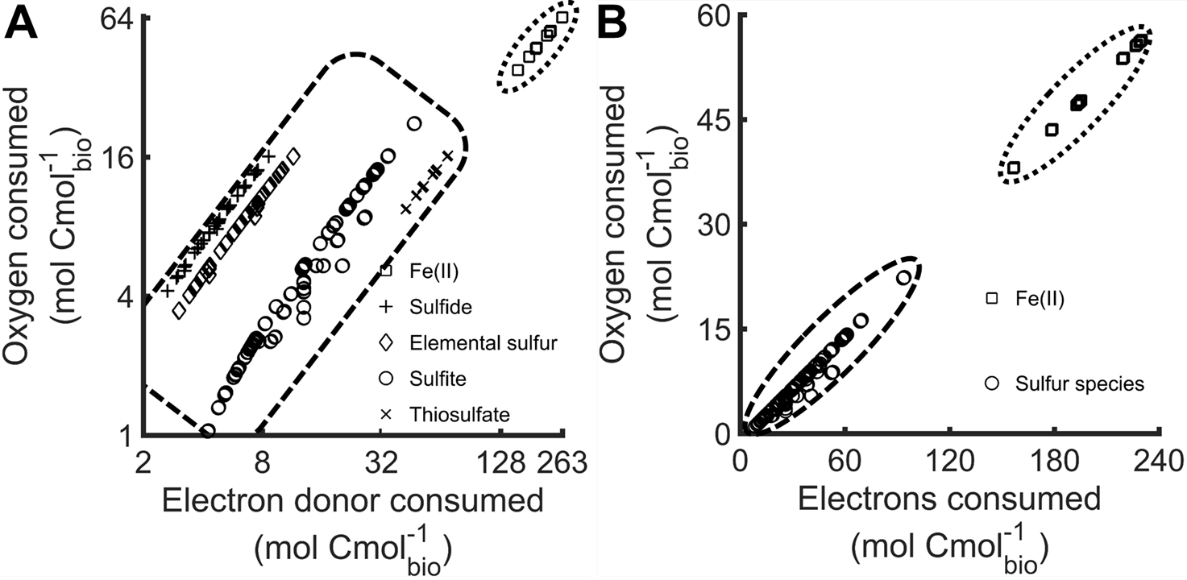


**Figure B.** Electron donor required to produce autotroph biomass. The oxygen consumption for all biomass producing elementary flux modes of *M. yellowstonensis* plotted as a function of mole electron donor (A) or mole electrons (B) consumed to produce a Cmole of biomass. Specific electron donors include Fe(II), sulfide, elemental sulfur, sulfite, and thiosulfate.

Determination of relative population abundance of autotroph to heterotroph

The relative abundance of autotroph to heterotroph was approximated by previously published sequence analyses (Table C) [8]. *Geoarchaeum* str. OSPB and Novel Archaeal Group 2 are believed to belong to similar functional guilds and their collective metabolic behavior was represented by the *Geoarchaeum* str. OSPB *in silico* model [9]. A relative population abundance of autotroph to heterotroph of 0.3 to 0.5 was used to include the range of both examined springs (Table D).

**Table C.** Distribution of populations in One Hundred Springs Plain (OSP) and Beowulf hot springs from 454 and Illumina sequence analyses.

|  | OSP | | |  | Beowulf | | |
| --- | --- | --- | --- | --- | --- | --- | --- |
| **Autotrophs** | 454 | Illumina | Averaged Percent |  | 454 | Illumina | Averaged Percent |
| *M. yellowstonensis* | 10.3 | 16 | 21% |  | 6.6 | 12 | 14% |
| *Hydrogenobaculum* | 3.4 | 3 | 5% |  | 1.2 | 4.3 | 4% |
| **Heterotrophs** |  |  |  |  |  |  |  |
| *Geoarchaeum* str. OSPB | 43.4 | 33 | 60% |  | 3 | 6 | 7% |
| Novel Archaeal Group 2 | 4 | 6 | 8% |  | 38 | 27 | 48% |
| **Others** |  |  |  |  |  |  |  |
| *Thaumarchaeota* | 0.4 | 0.4 | 1% |  | 4.4 | 2 | 5% |
| Novel Archaeal Group 3 | 0.1 | 0.12 | 0% |  | 3 | 5 | 6% |
| Other *Sulfolobales* | 2.5 | 4 | 5% |  | 10 | 11 | 16% |
| *Nanoarchaeota* | 0.2 | 0.6 | 1% |  | 0.1 | 0.6 | 1% |
| **Total** | 64.3 | 63.12 | 100% |  | 66.3 | 67.9 | 100% |

Modified from [8].

**Table D.** Relative population abundance of autotroph to heterotroph considering different representative populations based on Table C.

|  | OSP | |  | Beowulf | |
| --- | --- | --- | --- | --- | --- |
| Calculation of Relative Population Abundance | 454 | Illumina |  | 454 | Illumina |
| MK1/Geo | 0.24 | 0.48 |  | 2.20 | 2.00 |
| MK1/(Geo+NAG2) | 0.22 | 0.41 |  | 0.16 | 0.36 |
|  |  |  |  |  |  |

Abbreviations are as follows: MK1 - *M. yellowstonensis*; Geo - *Geoarchaeum* str. OSPB; NAG2 - Novel Archaeal Group 2;

Additional limits for total biomass concentrations in the mat community


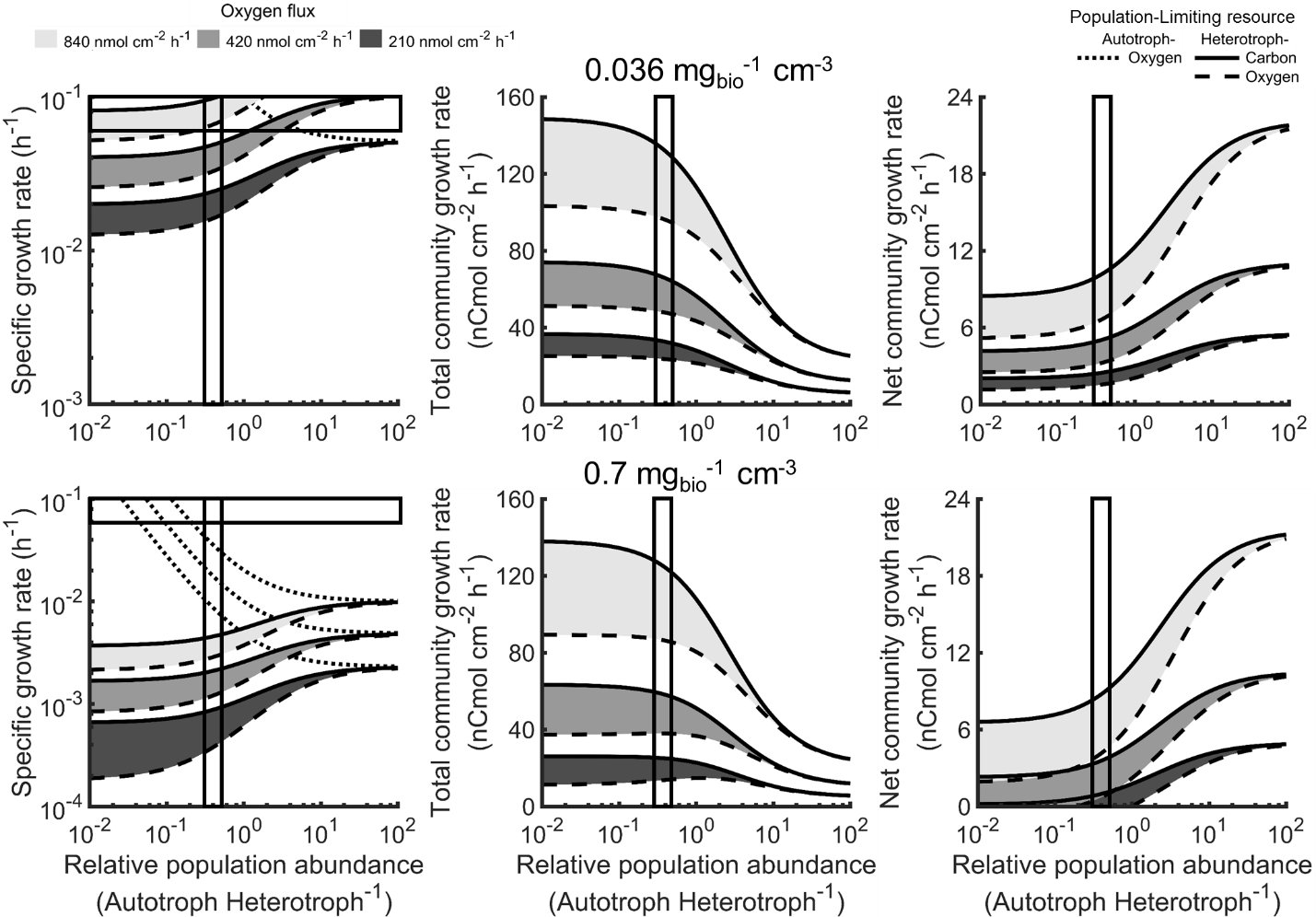


**Figure C.** Overlaying simulation of various nutrient limitation, relative population abundance, and *in situ* parameters established limits for the total biomass concentration in a mat community. Three oxygen fluxes, representing 50, 100, and 200 %, of the average observed oxygen flux (420 nmol O_2_ cm^-2^ h^-1^) provided upper and lower bounds of resource availability [8,10]. An autotroph community with 100% oxygen flux was limited by the growth rate of the autotroph at a total biomass concentration of 0.036 mg cm^-3^. A heterotroph community with 100% oxygen flux became limited by oxygen flux at a total biomass concentration of 0.7 mg cm^-3^. Each modeled oxygen flux was analyzed using the predicted most efficient metabolic strategy for utilization of oxygen (solid line) and carbon (dashed line). A constant aerobic volume allowed for the prediction of specific growth rates. The predicted relative population abundance of 0.3 to 0.5 autotroph to heterotroph is shown by the vertical dashed lines.

Graphical representations of metabolic space simulated using hybrid EFM-FBA


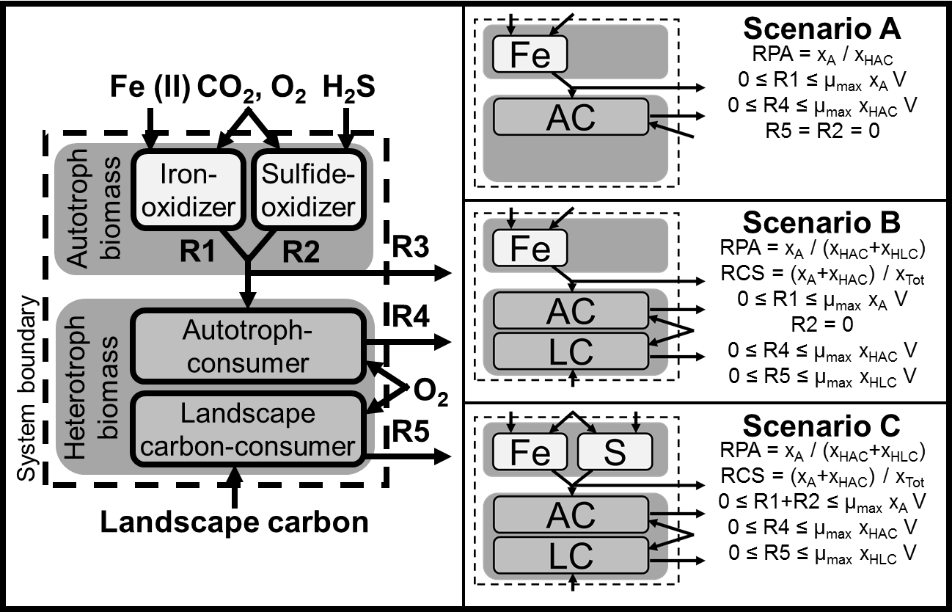


**Figure D.** Conceptual representation and associated parametric bounds of sensitivity analysis. Abbreviations: R1 or Fe, biomass produced by the iron-oxidizing autotroph; R2 or S, biomass produced by the sulfide-oxidizing autotroph; R3, net biomass produced by both autotrophs; R4 or AC, biomass produced by the autotroph-consuming heterotroph; R5 or LC, biomass produced by the landscape carbon-consuming heterotroph; RPA, relative population abundance; and RCS, relative carbon source. All other parameters are as described in Table 1.


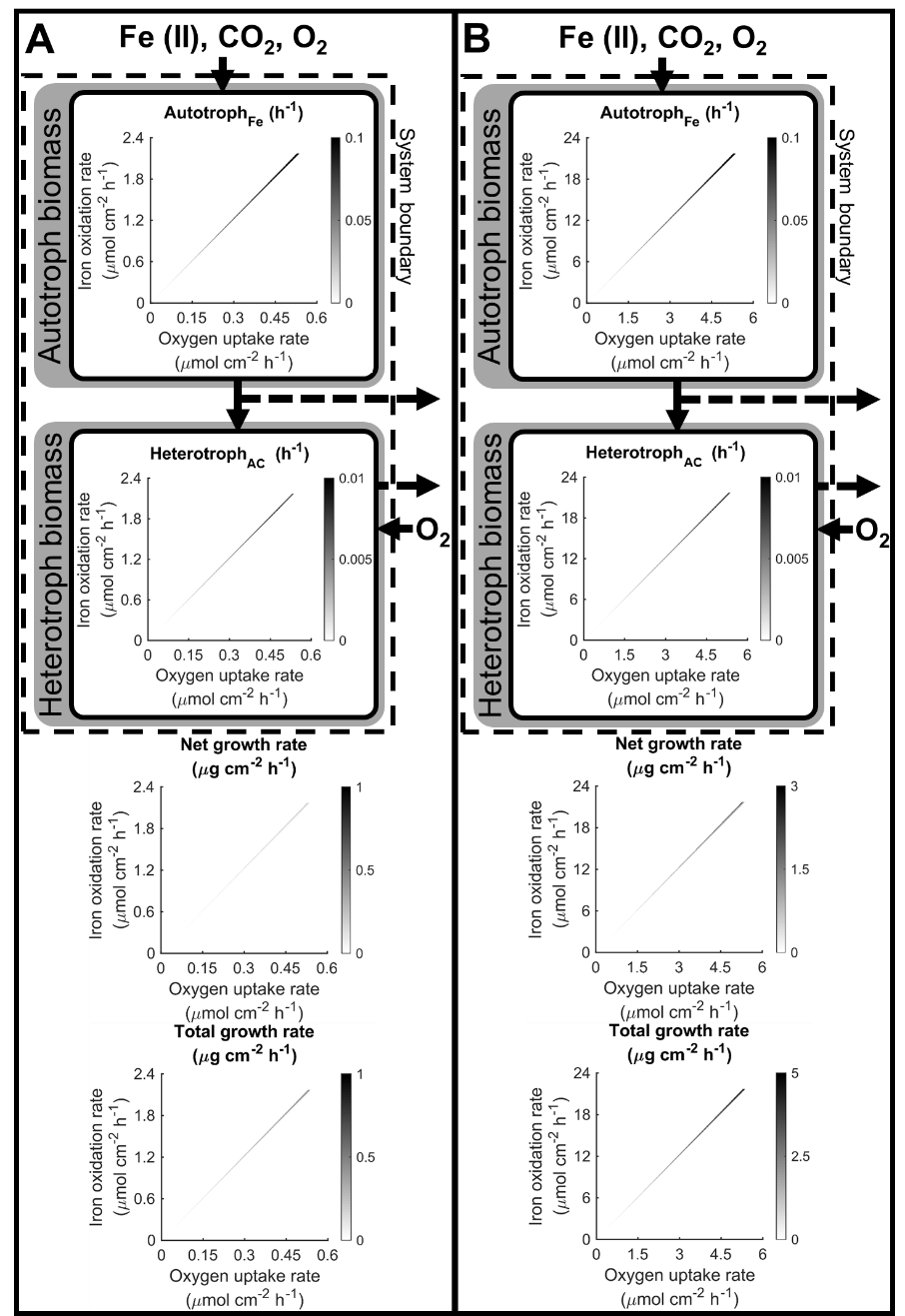


**Figure E.** Metabolic space feasible for a community composed of an iron oxidizing autotroph and autotroph consuming heterotroph. Active biomass concentrations were 0.2 (A) and 2.0 (B) mg cm^-3^ and an aerobic volume of 0.07 cm^3^ cm^-2^ allowed for the prediction of specific growth rates under the examined conditions bounding the possible physiological space assuming a maximum growth rate of 0.1 hr^-1^. The observed relative population abundances were 0.3 to 0.5 based on sequence analyses.


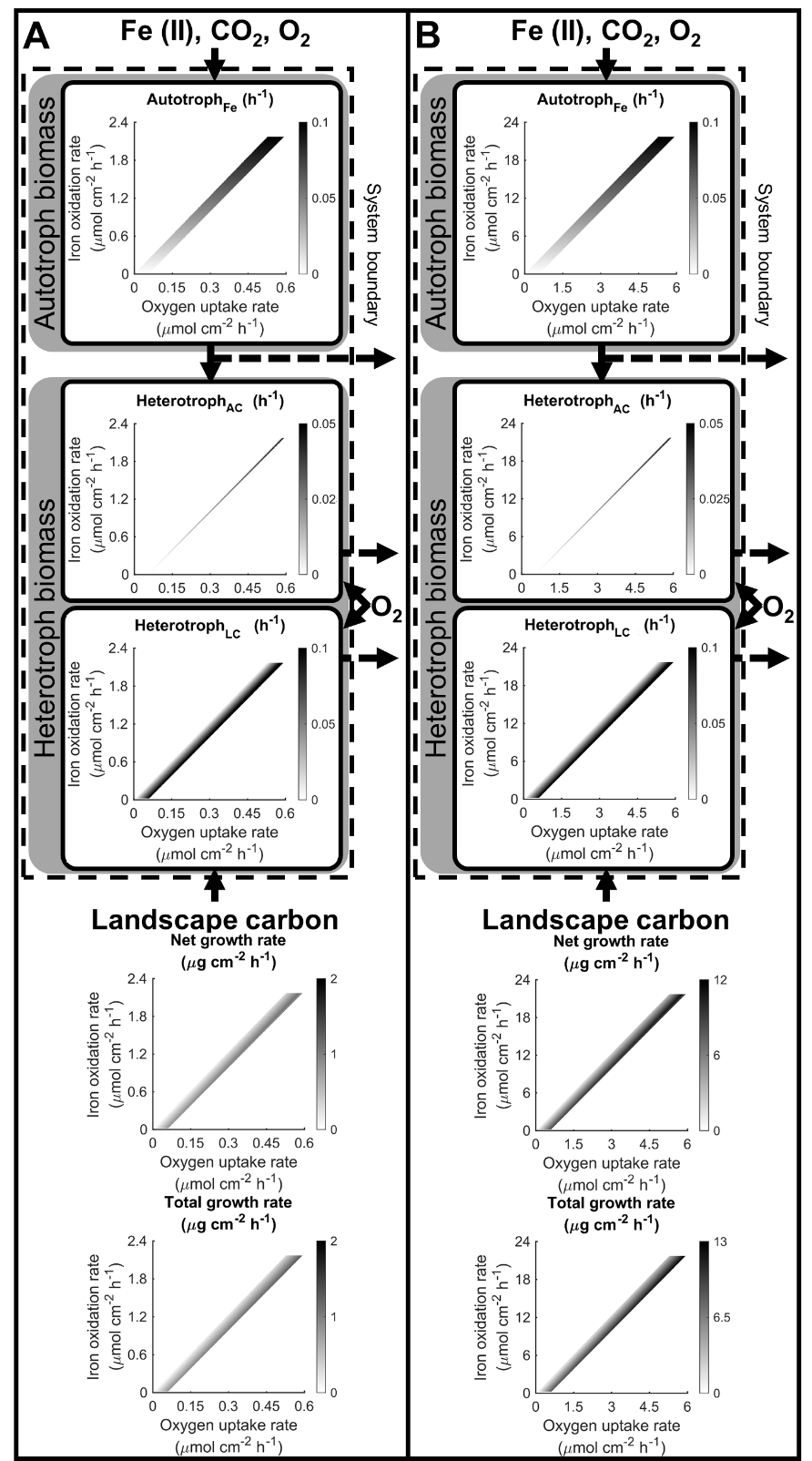


**Figure F.** Metabolic space feasible for a community composed of an iron oxidizing autotroph, an autotroph consuming heterotroph and a landscape carbon consuming heterotroph. Active biomass concentrations were 0.2 (A) and 2.0 (B) mg cm^-3^ and an aerobic volume of 0.07 cm^3^ cm^-2^ allowed for the prediction of specific growth rates under the examined conditions bounding the possible physiological space assuming a maximum growth rate of 0.1 hr^-1^. The observed relative population abundances were 0.3 to 0.5 based on sequence analyses. A maximum of 58 % of mat biomass carbon could be of landscape carbon origins.


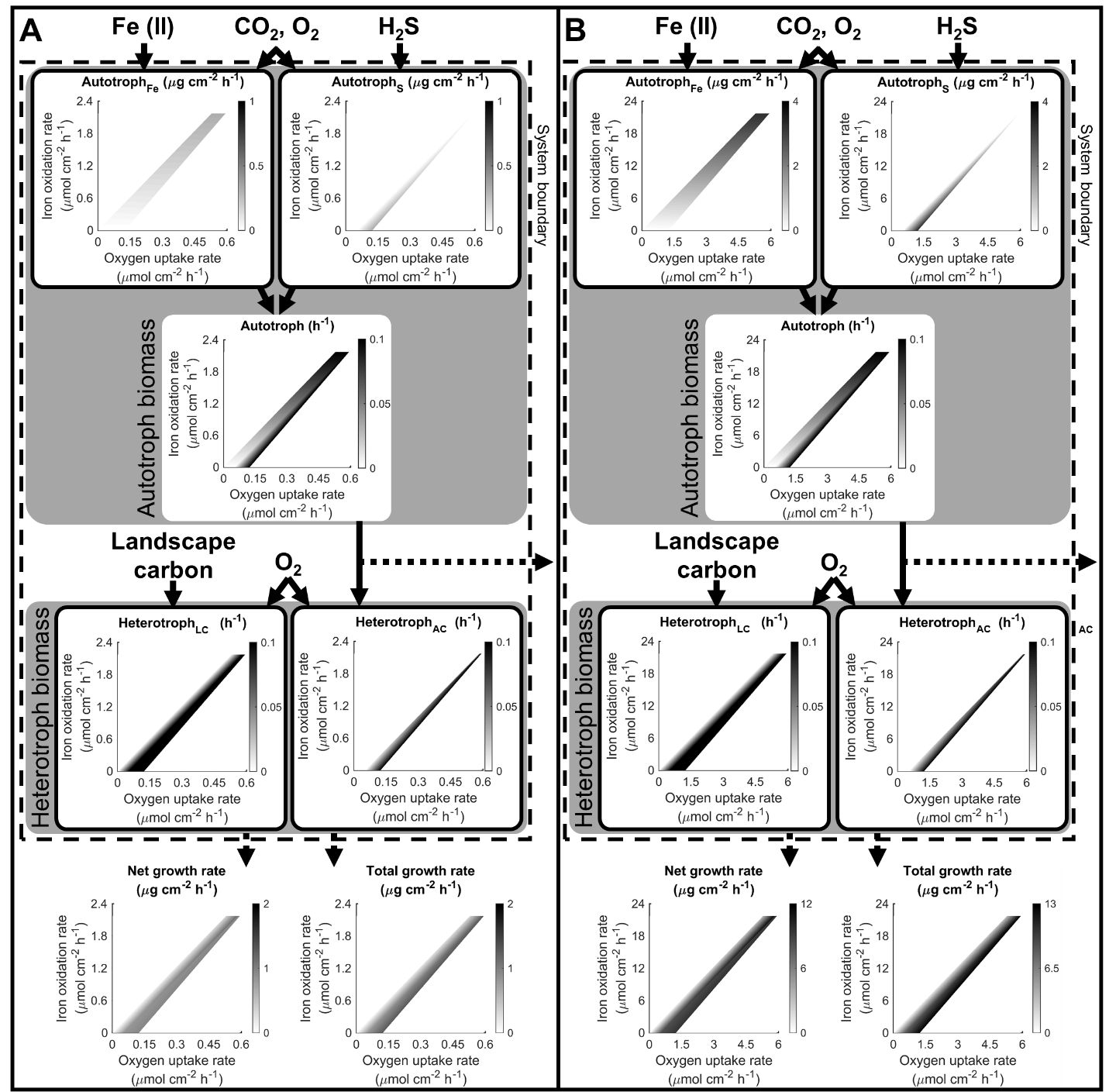


**Figure G.** Metabolic space feasible for a community composed of an iron oxidizing autotroph, a sulfur oxidizing autotroph, an autotroph consuming heterotroph and a landscape carbon consuming heterotroph. Active biomass concentrations were 0.2 (A) and 2.0 (B) mg cm^-3^ and an aerobic volume of 0.07 cm^3^ cm^-2^ allowed for the prediction of specific growth rates under the examined conditions bounding the possible physiological space assuming a maximum growth rate of 0.1 hr^-1^. The observed relative population abundances were 0.3 to 0.5 based on sequence analyses. A maximum of 58 % of mat biomass carbon could be of landscape carbon origins. The total specific growth rate of autotroph was bound allowing complete iron oxidation or complete sulfide oxidation and any combination thereof since the ratio of the two was not known.

Autotroph biomass turnover to sustain the heterotrophic population


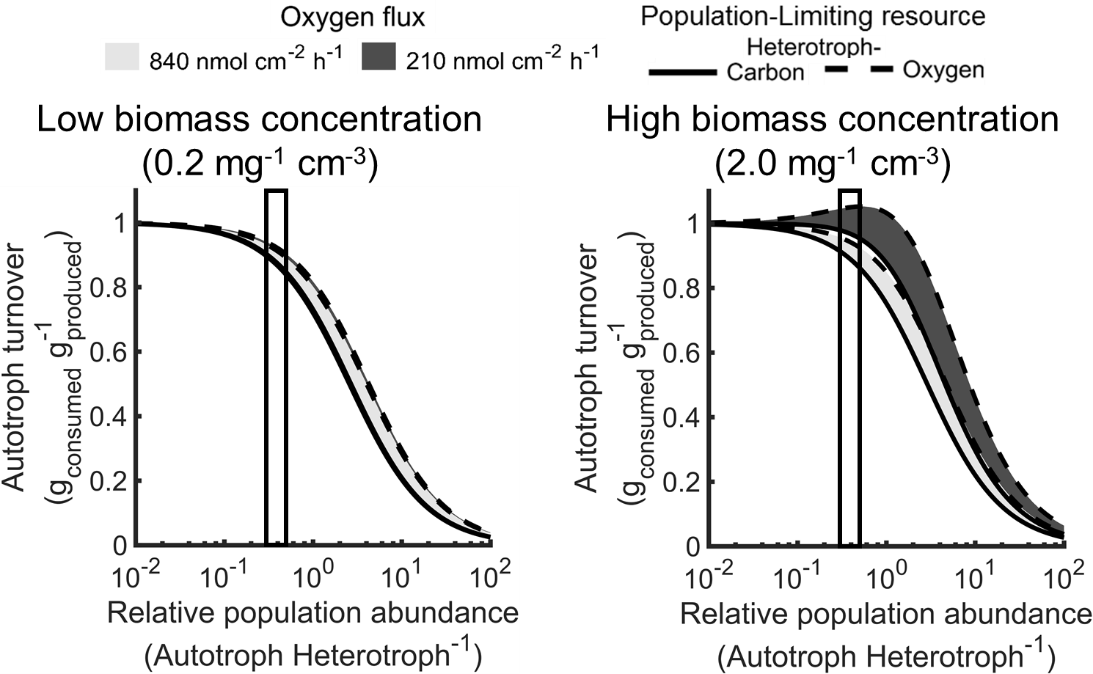


**Figure H.** Increasing relative population abundance of heterotroph required an increase in turnover of autotroph biomass, at the high biomass concentration requiring more autotroph than produced. The observed relative abundance of 0.3 – 0.5 required that at least 80% of the autotroph produced be consumed by the heterotroph. Both oxygen fluxes resulted in similar turnovers for the low biomass concentration simulation.

Sensitivity analysis of nongrowth associated maintenance energy


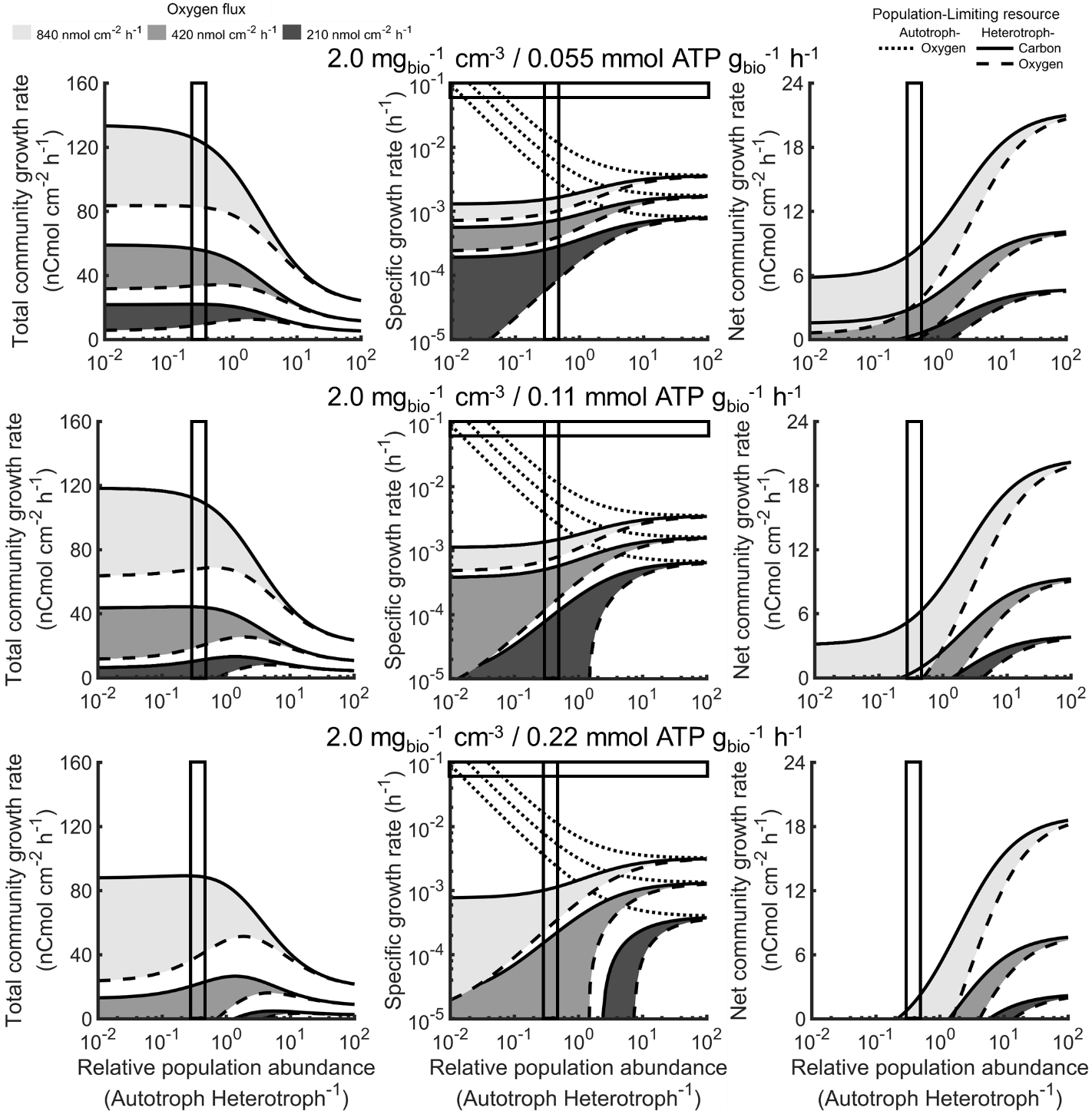


**Figure I.** Effect of nongrowth associated maintenance energy (NGAM) on community growth. Three values for NGAM were examined that represented 50, 100, and 200 % of the calculated value (row) [11]. Three oxygen fluxes were examined that represented 50, 100, and 200 %, respectively, of the average observed oxygen flux (420 nmol O_2_ cm^-2^ h^-1^) [8,10]. Each modeled oxygen flux was analyzed using the predicted most efficient metabolic strategy for oxygen (solid line) and carbon (dashed line). Active biomass concentration was 2.0 mg cm^-3^ and an aerobic volume of 0.07 cm^3^ cm^-2^ allowed for the prediction of specific growth rates under the examined conditions. The observed relative population abundance was 0.3 and 0.5 (vertical dashed lines) based on sequence analyses. A biomass concentration of 1.0 mg cm^-3^ and NGAM of 0.22 mmol ATP g_bio_^-1^ h^-1^ gave the same results as 2.0 mg cm^-3^ and NGAM of 0.11 mmol ATP g_bio_^-1^ h^-1^. The effect of NGAM was negligible for the autotroph at a growth rate of 0.1 h^-1^.

Determination of total biomass concentration from *in situ* Fe measurements

The total biomass concentrations of OSP and Beowulf springs were approximated from the Fe deposition rate using the elemental composition of Fe(III)-oxide mats. Fe-oxide deposition rates of 8.73 and 1.28 µmol_Fe_ cm^-2^ d^-1^ at OSP and Beowulf springs were determined from previous characterization studies [8] assuming a constant deposition rate in a mature Fe(III)-oxide mat (i.e. older than 40 days) (Figure B) [8]. Total biomass concentrations were extrapolated using the elemental composition of the mats and density as derived from the reported vertical growth rate of the Fe(III)-oxide mat (Table E).

**Table E.** Biomass concentration calculations based on Fe-oxide deposition rate.

| Description | OSP | Beowulf | Units |
| --- | --- | --- | --- |
| Fe deposition rate | 8.73 | 1.28 | µmol_Fe_ cm^-2^ d^-1^ |
| Fe concentration across mat growth rates ^a^ | 0.24 | 0.036 | g_Fe_ cm^-3^ |
| Mat carbon per mat iron ^b^ | 0.046 | 0.046 | g_CTot_ g_Fe_^-1^ |
| Biomass carbon per total mat carbon ^c^ | 0.1 | 0.1 | g_CBio_ g_CTot_^-1^ |
| Carbon per gram of biomass from the model ^d^ | 0.043 | 0.043 | Cmol g_Bio_^-1^ |
| Biomass concentration | 2.15 | 0.32 | mg_Bio_ cm^-3^ |

^a^: An average vertical growth rates of 22.5 µm cm^-2^ d^-1^ was based on [8]. ^b^: [12]. ^c^: [13]. ^d^: [5].


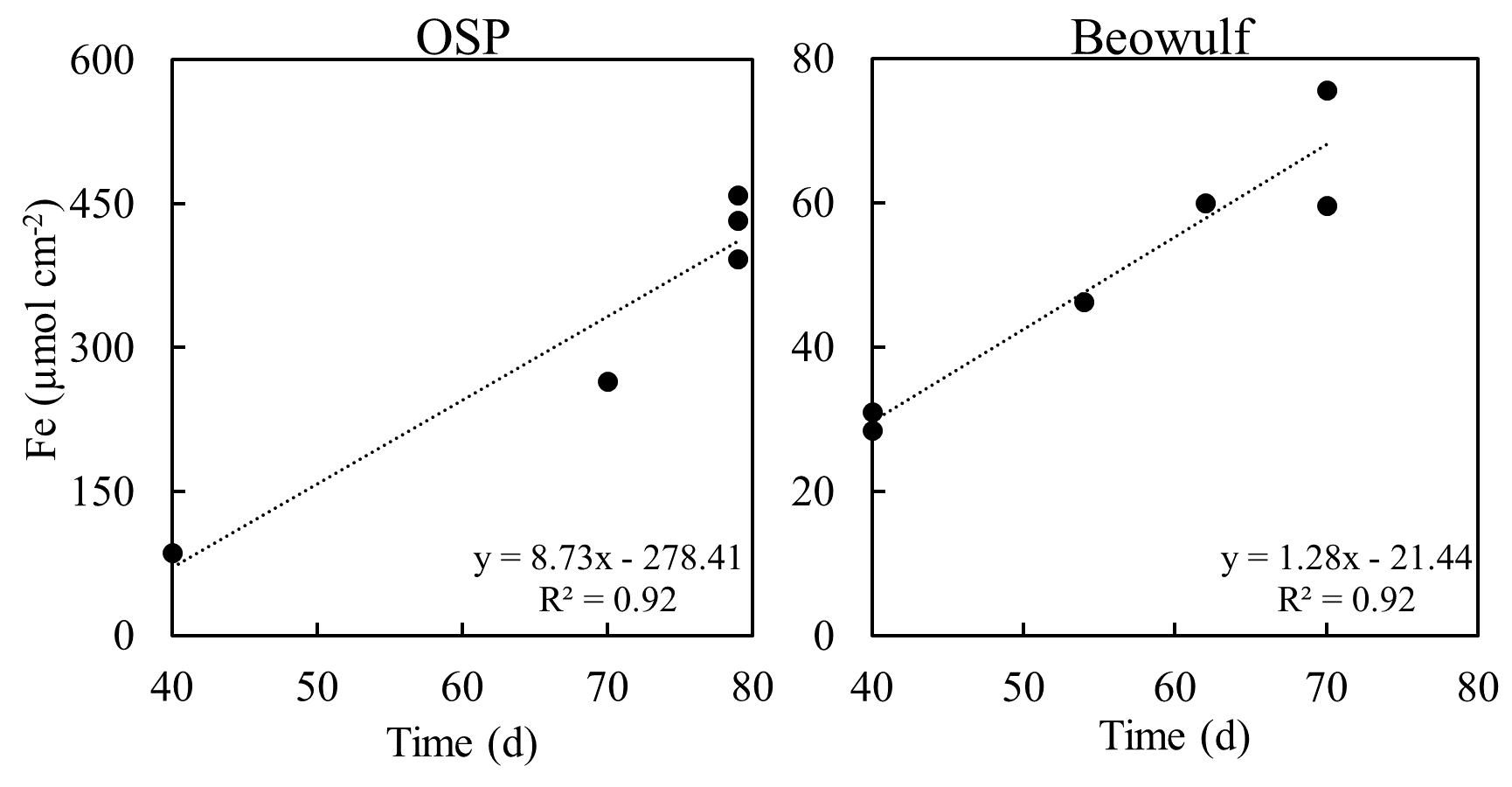


**Figure J.** Fe-oxide deposition rate for One Hundred Springs Plain (OSP) and Beowulf hot springs. A linear approximation was used to determine the deposition rate under the assumption that a developed mat would have constant microbial activity in the oxic zone. Data from [8].

Impact of metabolite exchange on the maximum and minimum biomass concentration


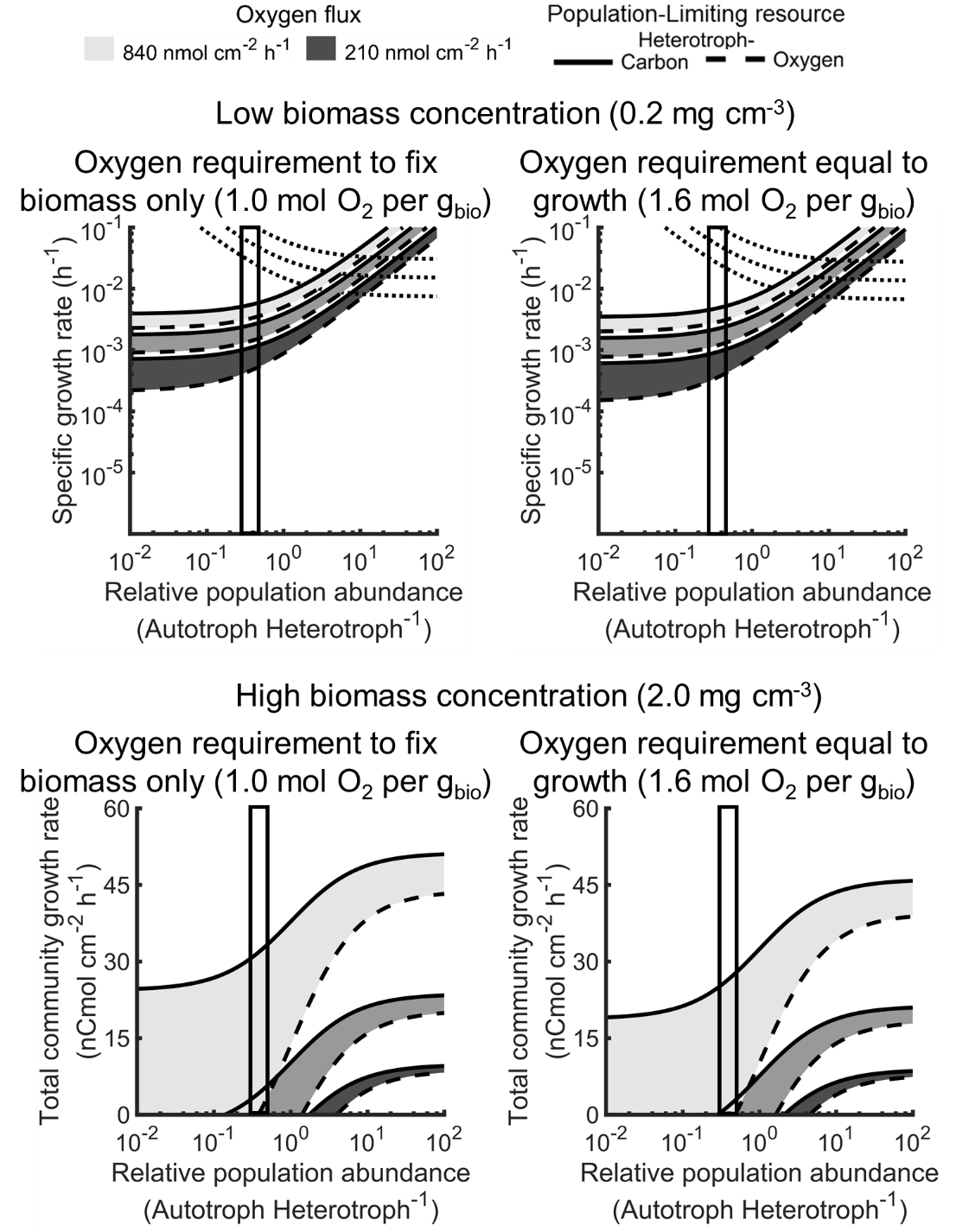


**Figure K.** Predicted maximum and minimum biomass concentrations were relatively unchanged by simulating metabolite exchange as opposed to lysis. Metabolite exchange was simulated by accounting for the carbon consumed by heterotroph as a lower oxygen requirement equal to that required to fix the carbon in autotroph biomass. The upper limit for this cost was represented as the oxygen required for autotroph biomass growth, which included polymerization and maintenance energy.

References

1. Price ND, Reed JL, Palsson BØ. Genome-scale models of microbial cells: evaluating the consequences of constraints. Nat Rev Microbiol. 2004;2: 886–97. doi:10.1038/nrmicro1023

2. Feist AM, Herrgård MJ, Thiele I, Reed JL, Palsson BØ. Reconstruction of biochemical networks in microorganisms. Nat Rev Microbiol. 2009;7: 129–43. doi:10.1038/nrmicro1949

3. Trinh CT, Wlaschin A, Srienc F. Elementary mode analysis: a useful metabolic pathway analysis tool for characterizing cellular metabolism. Appl Microbiol Biotechnol. 2009;81: 813–826. doi:10.1007/s00253-008-1770-1

4. Beck AE, Hunt KA, Bernstein HC, Carlson RP. Interpreting and designing microbial communities for bioprocess applications, from components to interactions to emergent properties. In: Eckert C, Trinh CT, editors. Biotechnology for Biofuel Production and Optimization. 1st ed. Amsterdam, Netherlands: Elsevier; 2016. pp. 407–432. doi:10.1016/B978-0-444-63475-7.00015-7

5. Hunt KA, Jennings R de M, Inskeep WP, Carlson RP. Stoichiometric modelling of assimilatory and dissimilatory biomass utilisation in a microbial community. Environ Microbiol. 2016;18: 4946–4960. doi:10.1111/1462-2920.13444

6. Kozubal M, Macur RE, Korf S, Taylor WP, Ackerman GG, Nagy A, et al. Isolation and distribution of a novel iron-oxidizing crenarchaeon from acidic geothermal springs in Yellowstone National Park. Appl Environ Microbiol. 2008;74: 942–949. doi:10.1128/AEM.01200-07

7. Kozubal MA, Dlakic M, Macur RE, Inskeep WP. Terminal oxidase diversity and function in “*Metallosphaera yellowstonensis*”: gene expression and protein modeling suggest mechanisms of Fe(II) oxidation in the Sulfolobales. Appl Environ Microbiol. 2011;77: 1844–1853. doi:10.1128/AEM.01646-10

8. Beam JP, Bernstein HC, Jay ZJ, Kozubal MA, Jennings R deM., Tringe SG, et al. Assembly and succession of iron oxide microbial mat communities in acidic geothermal springs. Front Microbiol. 2016;7: 25. doi:10.3389/fmicb.2016.00025

9. Kozubal MA, Macur RE, Jay ZJ, Beam JP, Malfatti SA, Tringe SG, et al. Microbial iron cycling in acidic geothermal springs of Yellowstone National Park: integrating molecular surveys, geochemical processes, and isolation of novel Fe-active microorganisms. Front Microbiol. 2012;3: 109. doi:10.3389/fmicb.2012.00109

10. Bernstein HC, Beam JP, Kozubal MA, Carlson RP, Inskeep WP. In situ analysis of oxygen consumption and diffusive transport in high-temperature acidic iron-oxide microbial mats. Environ Microbiol. 2013;15: 2360–2370. doi:10.1111/1462-2920.12109

11. Tijhuis L, Van Loosdrecht MCM, Heijnen JJ. A thermodynamically based correlation for maintenance gibbs energy requirements in aerobic and anaerobic chemotrophic growth. Biotechnol Bioeng. 1993;42: 509–519. doi:10.1002/bit.260420415

12. Inskeep WP, Macur RE, Harrison G, Bostick BC, Fendorf S. Biomineralization of As(V)-hydrous ferric oxyhydroxide in microbial mats of an acid-sulfate-chloride geothermal spring, Yellowstone National Park. Geochim Cosmochim Acta. 2004;68: 3141–3155. doi:10.1016/j.gca.2003.09.020

13. Jennings RM, Whitmore LM, Moran JJ, Kreuzer HW, Inskeep WP. Carbon dioxide fixation by *Metallosphaera yellowstonensis* and acidothermophilic iron-oxidizing microbial communities from Yellowstone National Park. Appl Environ Microbiol. 2014;80: 2665–2671. doi:10.1128/AEM.03416-13
